# Constitutive androgen receptor activity sustains DNA replication programs in triple-negative breast cancer

**DOI:** 10.1038/s41419-026-09180-4

**Published:** 2026-08-06

**Authors:** Jing Li, Hongwei Yuan, Kefan Chen, Jian Peng, Wu Du, Xinghai Li

**Affiliations:** Hinova Pharmaceuticals Inc, Chengdu, China

**Keywords:** Target validation, Prostate cancer

## Abstract

Triple-negative breast cancer (TNBC) is an aggressive subtype lacking effective targeted therapies. Despite AR expression in nearly half of TNBCs, androgen receptor signaling inhibitors (ARSIs) have shown minimal clinical benefit. In this study, we utilized HP518, a potent PROTAC that selectively degrades AR, to interrogate the role of AR in TNBC. We uncover a non-canonical, androgen-independent function of AR in AR-positive TNBC cells: AR cooperates with transcriptional regulators FoxA1 and GATA3 to promote expression of DNA replication genes critical for tumor proliferation. Genetic and pharmacological degradation of AR suppresses tumor cell growth, downregulates key DNA replication genes, and disrupts AR-associated transcriptional complexes. In AR-positive TNBC patient-derived xenografts (PDX), HP518 robustly inhibits tumor growth and synergizes with PI3Kα inhibition and chemotherapy. Our findings identify a previously unappreciated mechanism of AR action in TNBC and support AR degradation-not antagonism as a rational therapeutic strategy.

**Figure:**
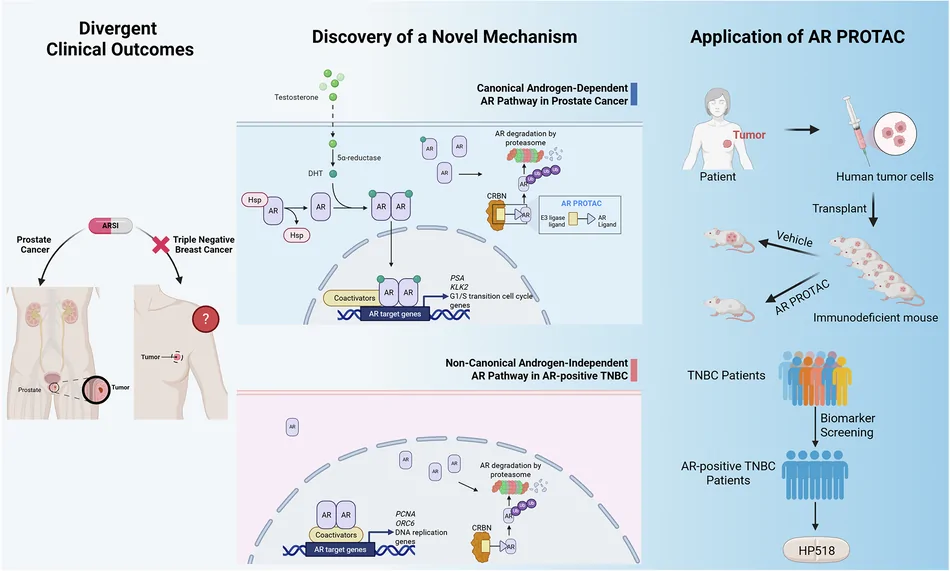

## Introduction

Triple-negative breast cancer (TNBC) is an aggressive and heterogeneous subtype of breast cancer characterized by the absence of estrogen receptor (ER), progesterone receptor (PR), and HER2 amplification. Accounting for 15–20% of breast carcinomas, TNBC disproportionately affects younger women and is characterized by high rates of recurrence, visceral metastasis, and poor overall survival [1, 2]. Unlike ER-positive or HER2-amplified tumors, which benefit from targeted therapies, TNBC treatment relies primarily on cytotoxic chemotherapy, yielding only modest long-term benefits. Although emerging modalities—such as immune checkpoint inhibitors and antibody–drug conjugates—have shown promise in select TNBC cohorts (e.g., PD-L1-positive or BRCA-mutant patients), most individuals still lack durable, biomarker-driven options. Therefore, identifying novel molecular targets remains a critical unmet need in TNBC management. The androgen receptor (AR) has emerged as a compelling target in a subset of TNBC, particularly the luminal androgen receptor (LAR) subtype, which expresses AR in 20–40% of cases and exhibits a transcriptional program driven by AR activity [3–5]. Preclinical studies have implicated AR in modulating proliferation, survival pathways, and resistance mechanisms in TNBC models, prompting clinical evaluation of AR signaling inhibitors (ARSIs) such as enzalutamide and bicalutamide. However, these trials yielded only modest response rates and failed to significantly prolong progression-free survival, indicating that competitive AR blockade does not fully abrogate AR’s oncogenic functions in TNBC [5, 6].

To dissect AR’s functional dependency in TNBC, we compared pharmacological inhibition with complete AR loss. siRNA-mediated silencing of AR in established AR-positive TNBC cell lines produced profound suppression of proliferation and viability, revealing that AR is essential for tumor cell growth independent of ligand stimulation. These findings suggested that AR protein degradation—rather than receptor antagonism—might more effectively eliminate AR’s pro-tumor activities and offer deeper mechanistic insight. Proteolysis-targeting chimeras (PROTACs) represent an innovative strategy to degrade target proteins via the ubiquitin–proteasome system. PROTAC molecules comprise a target-binding ligand, an E3 ubiquitin ligase recruiter, and a linker that facilitates formation of a ternary complex, leading to ubiquitination and proteasomal degradation of the target [7, 8]. This strategy removes both the enzymatic and non-enzymatic roles of the target, helps circumvent resistance driven by protein overexpression or mutation, and achieves durable suppression by routing the protein for proteasomal degradation.

In this study, we applied HP518, a clinical-stage AR PROTAC developed by Hinova Pharmaceuticals, to selectively degrade AR in AR-positive TNBC models. We demonstrate that AR’s classical androgen-dependent signaling is dispensable in these cells; instead, AR drives a distinct transcriptional program—collaborating with coactivators FoxA1 and GATA3—to regulate DNA replication genes critical for TNBC proliferation. HP518 is under clinical investigation for metastatic castration-resistant prostate cancer (NCT05252364, NCT06155084) and has received FDA Fast Track designation for inoperable or metastatic AR-positive TNBC. Our findings validate targeted AR degradation as a promising therapeutic strategy and illuminate a novel mechanistic paradigm for AR in TNBC.

## Results

### The androgen-dependent activity of AR is dispensable for the cell growth of AR-positive TNBC cells

Androgen receptor signaling inhibitors (ARSIs) are established therapies for prostate cancer; however, they have shown limited clinical benefit in patients with AR-positive triple-negative breast cancer (TNBC). To evaluate the role of AR in supporting the growth of AR-positive TNBC cells, we silenced AR expression using siRNAs and assessed cell proliferation in MDA-MB-453 cells—a well-characterized model of the luminal androgen receptor (LAR) subtype of TNBC, in which AR is a key oncogenic driver. AR depletion led to a complete inhibition of cell proliferation and a substantial reduction in cell survival (Fig. 1A).

**Fig. 1:**
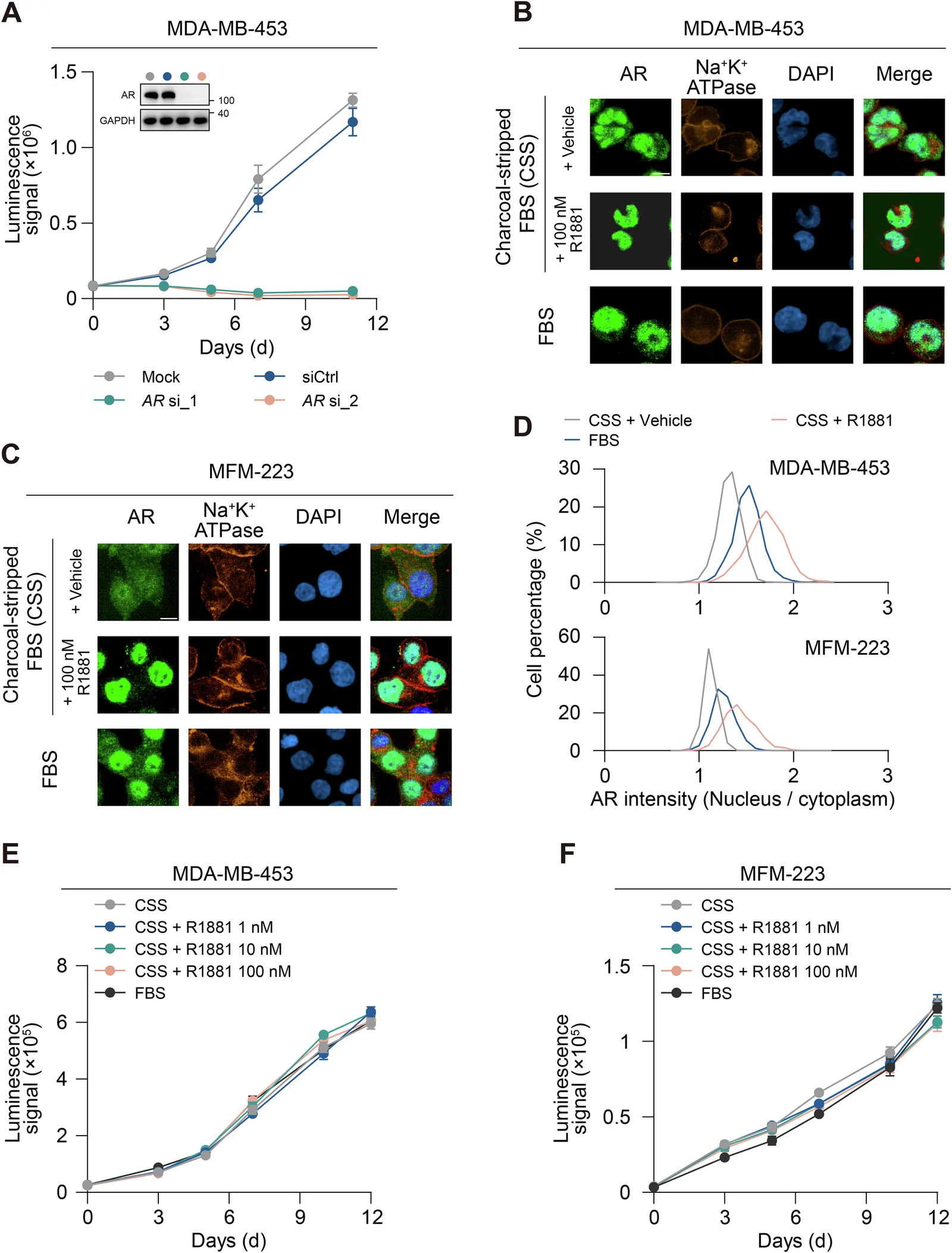
The androgen-dependent activity is dispensable for the cell growth of AR-positive TNBC. **A** Proliferation analysis of MDA-MB-453 cells transduced with mock, control siRNA, or *AR* siRNAs. Error bars, standard deviation (SD), *n* = 3. Inset: immunoblots of AR in MDA-MB-453 cells transduced with the indicated siRNAs for 48 h. **B**, **C** Immunofluorescence images of AR in MDA-MB-453 (**B**) and MFM-223 (**C**) cells cultured in medium supplemented with FBS or charcoal-stripped FBS (CSS) in the presence or absence of R1881. Scale bar, 10 μm. **D** Quantifications of AR distribution in MDA-MB-453 and MFM-223 cells from (**B**) and (**C**). Proliferation analysis of MDA-MB-453 (**E**) and MFM-223 (**F**) cells cultured in medium supplemented with FBS or CSS in the presence or absence of R1881. Data expressed as mean ± SD (*n* = 3).

To assess whether androgen influences AR subcellular localization in TNBC, we examined AR distribution in two AR-positive cell lines, MDA-MB-453 and MFM-223. In both models, AR was predominantly nuclear at baseline; stimulation with 100 nM R1881 (a synthetic androgen) increased nuclear AR signal intensity (Fig. 1B–D). Notably, further biochemical fractionation revealed that this increase in nuclear AR was not consistently accompanied by a reduction in the cytoplasmic fraction, and only a modest change in total AR protein abundance was observed (Fig. S1), indicating that the enhanced nuclear signal cannot be solely ascribed to nucleocytoplasmic redistribution of the pre-existing cytoplasmic pool.

Despite this ligand-induced nuclear accumulation of AR, R1881 treatment failed to elicit a marked proliferative response in these AR-positive TNBC cells (Fig. 1E, F). Together, these findings indicate that while AR protein dynamics remain functionally responsive to androgen stimulation, TNBC cell growth does not depend on androgen signaling, implying the existence of an androgen-independent mechanism underpinning AR-positive TNBC survival.

### Targeted degradation of AR impairs cell proliferation in AR-positive TNBC

To further explore the therapeutic potential of targeting AR in AR-positive TNBC, we employed AR PROTACs to degrade AR via the ubiquitin–proteasome pathway, rather than using siRNA-mediated knockdown. We found that HP518, a selective AR PROTAC, effectively induced AR degradation via the ubiquitin–proteasome pathway at concentrations as low as 1 nM without notable off-target effects (Fig. 2A, Fig. S2). Notably, Western blot analysis revealed that HP518 exhibits significantly greater AR degradation activity compared to ARV-110 and ARV-766, two AR-targeting PROTACs currently in clinical development by Arvinas (Fig. 2B, C). Next, we assessed the anti-proliferative efficacy of AR PROTACs versus androgen receptor signaling inhibitors (ARSIs)—namely enzalutamide and HC-1119 (a deuterated enzalutamide analog developed by Hinova Pharmaceuticals Inc.)—both of which competitively block androgen binding to AR [9]. HP518 markedly reduced viability in AR-positive TNBC cells, outperforming the clinical PROTACs ARV-110 and ARV-766, while exhibiting negligible activity in AR-negative TNBC lines MDA-MB-468 and HCC1937 (Fig. 2D–F). Consistently, colony formation assays further confirmed that AR depletion significantly impaired long-term clonogenic survival in AR-positive TNBC cells (Fig. S3). In contrast, ARSIs had only minimal impact on the proliferation of AR-positive TNBC models (Fig. 2G), consistent with prior clinical observations of limited ARSI efficacy in this setting. Given that ARV-110 and ARV-766 exhibited weaker anti-proliferative activity—likely due to their relatively lower AR degradation potency compared to HP518—these results suggest that AR plays a critical role in TNBC cell survival, and that the extent of AR degradation by PROTACs is positively correlated with their anti-proliferative efficacy. Notably, HP518 maintained its anti-proliferative activity in charcoal-stripped medium, and supplementation with androgen did not alter this effect (Fig. S4), further supporting the notion that AR contributes to TNBC cell growth through an androgen-independent mechanism.

**Fig. 2:**
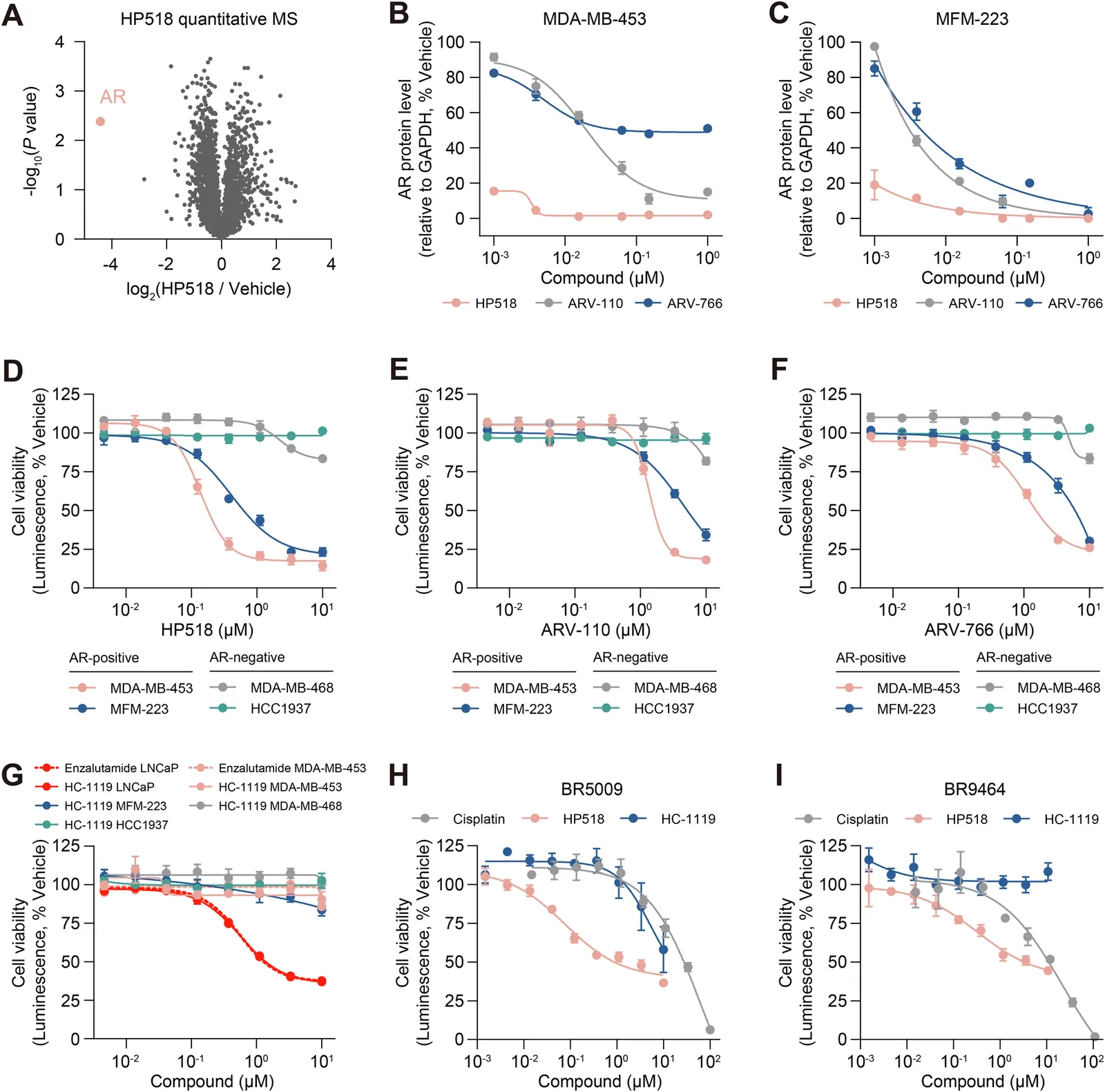
Targeted degradation of AR impairs cell fitness of AR-positive TNBC. **A** Quantitative mass spectrometry of VCaP cells treated with vehicle or 10 nM HP518 for 6 h. *n* = 3. Quantifications of immunoblots of AR in MDA-MB-453 (**B**) and MFM-223 (**C**) cells treated with the indicated compounds for 24 h. *n* = 3. Drug sensitivities of AR-positive (MDA-MB-453 and MFM-223) cells and AR-negative (MDA-MB-468 and HCC1937) cells after 5 days of treatment with HP518 (**D**), ARV-110 (**E**), or ARV-766 (**F**). Error bars, SD, *n* = 3. **G** Drug sensitivities of the indicated cells after 5 days of treatment with enzalutamide or HC-1119, a deuterated enzalutamide. Error bars, SD, *n* = 3. **H**, **I** Drug sensitivities of BR5009 and BR9464 cells derived from AR-positive TNBC patients after 7 days of treatment with cisplatin, HP518, or HC-1119. Data expressed as mean ± SD (*n* = 3).

We next extended our analysis to AR-positive patient-derived TNBC samples, BR5009 and BR9464 (Fig. S5). As anticipated, HP518 markedly impaired cell viability in both samples (Fig. 2H, I), reinforcing the relevance of AR degradation in this setting. Collectively, these findings suggest that the canonical androgen-dependent AR signaling pathway may be dispensable in AR-positive TNBC. Instead, our results support a model in which AR exerts a critical androgen-independent role in maintaining TNBC cell survival and proliferation, pointing to a novel AR-driven pathway distinct from its classical role in androgen signaling.

### AR regulates DNA replication genes in AR-positive TNBC cells

In prostate cancer cells, AR acts as a transcription factor that promotes tumor progression in both androgen-dependent and -independent manners, and the AR-regulated genes have been extensively studied and defined. To examine whether these genes are also regulated by AR in AR-positive TNBC cells, MDA-MB-453 cells were treated with the AR degrader HP518 or the AR inhibitor HC-1119 (Fig. S6A). Neither treatment affected the expression of androgen-dependent (Fig. S6B, top) and -independent (Fig. S6C) AR-regulated genes in TNBC cells, while markedly downregulating canonical androgen-responsive genes in LNCaP prostate cancer cells (Fig. S6B, bottom), suggesting that AR promotes cell proliferation in TNBC via a different mechanism.

To further explore the underlying mechanism, we performed RNA sequencing (RNA-Seq) to evaluate transcriptomic changes following treatment of MDA-MB-453 cells with HP518 or HC-1119. We identified 326 significantly downregulated genes and 437 upregulated genes in HP518-treated cells, while HC-1119 treatment resulted in 34 downregulated and 32 upregulated genes (Fig. S7A, B, Table S1). Gene ontology analyses revealed that downregulated genes induced by HP518 were involved in DNA replication and double-strand break repair signaling pathways, differing from findings in prostate cancer (Fig. 3A). The upregulated genes in HP518-treated cells were predominantly enriched in apoptotic-related pathways (Fig. S7C). In contrast, genes affected by HC-1119 did not show significant enrichment in any gene sets (Fig. S7D, E). Notably, a reduction in the expression of DNA replication genes (e.g., *PCNA*, *ORC6*) was found only in the HP518-treated group (Fig. 3B).

**Fig. 3:**
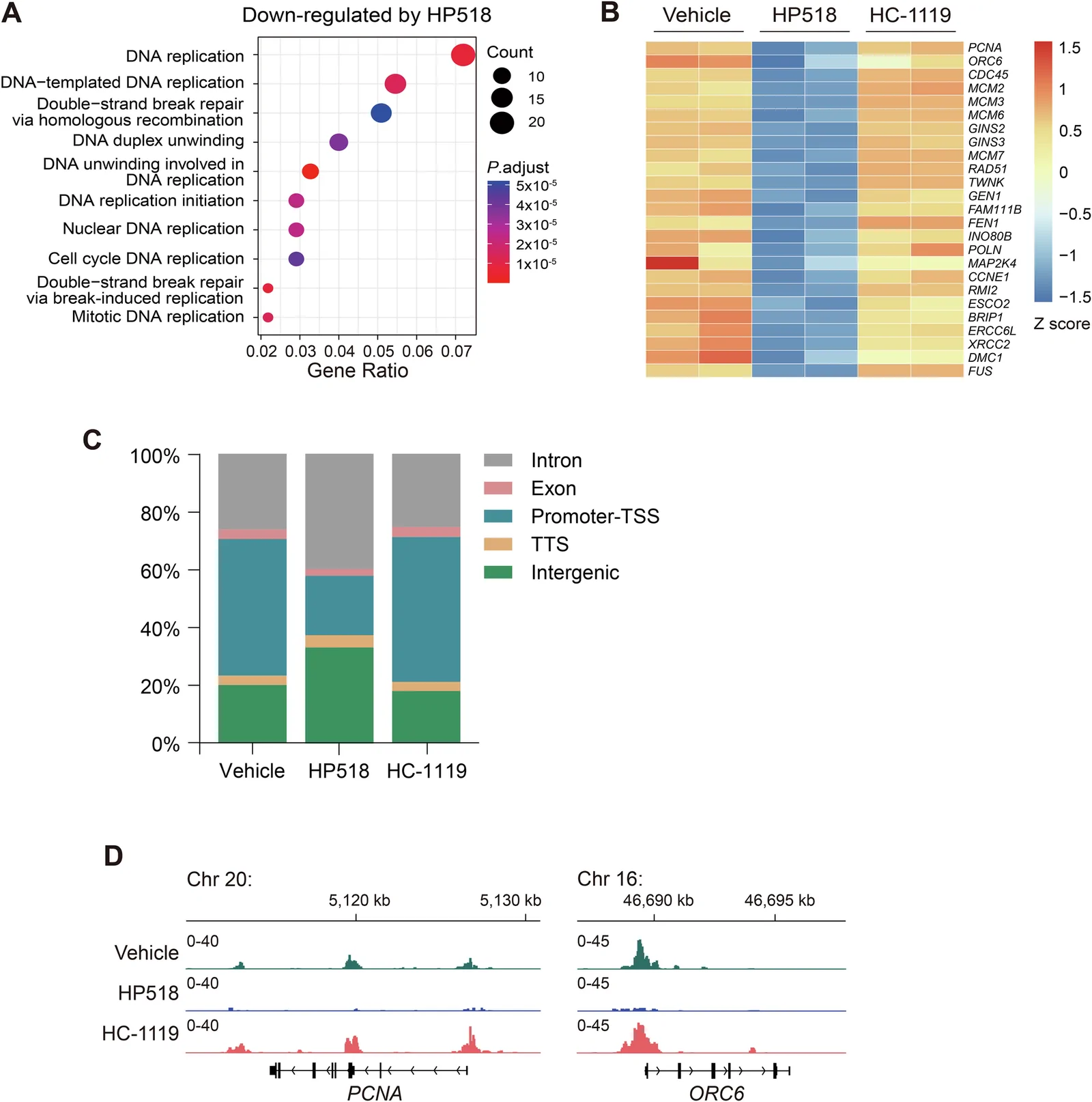
DNA replication pathway is disturbed after AR downregulation. **A** Gene Ontology enrichment analysis of differentially downregulated genes in MDA-MB-453 cells treated with 100 nM HP518 versus vehicle for 24 h. *n* = 2. Genes differentially downregulated were defined as log_2_(HP518/vehicle) < −1 and *P*-value < 0.01. **B** Heat map of HP518-regulated DNA replication-related genes in MDA-MB-453 cells treated with vehicle, 100 nM HP518, or 1 μM HC-1119. A row-wise Z-score transformation was performed. *n* = 2. **C** Genomic annotations of indicated classes of CUT&Tag peaks. *n* = 2. **D** AR CUT&Tag tracks showing changes in chromatin accessibility at the *PCNA* and *ORC6* loci in MDA-MB-453 cells treated with vehicle, 100 nM HP518, or 1 μM HC-1119 for 8 h. *n* = 2.

To investigate how AR regulates the transcription of DNA replication genes, we employed Cleavage Under Targets and Tagmentation (CUT & Tag) [10] to profile AR–chromatin accessibility in MDA-MB-453 cells treated with HP518 or HC-1119. Our analysis revealed significant peaks, with HP518 significantly reducing peak enrichment at the promoter-transcription start site (TSS) compared to the vehicle (DMSO), while HC-1119 had no effect (Fig. 3C). Notably, HP518 impaired AR occupancy at the promoters of *PCNA* and *ORC6*, while HC-1119 had no effect (Fig. 3D). Consistent with our earlier observations (Fig. S6B, C), canonical AR target genes defined in prostate cancer were neither prominently occupied by AR nor regulated by HP518 or HC-1119 in MDA-MB-453 cells. Together, these findings indicate that AR preferentially regulates DNA replication gene programs in TNBC, including genes such as *PCNA* and *ORC6*, distinct from its canonical transcriptional role in prostate cancer.

### Expression of PCNA and ORC6 is positively correlated with AR

Proliferating cell nuclear antigen (PCNA) and origin recognition complex subunit 6 (ORC6) are key factors involved in DNA replication [11–13]. To confirm that the expression of these genes is indeed regulated by AR, MDA-MB-453 cells were treated with the *AR*-specific siRNAs or AR degrader HP518. Both treatments resulted in robust reductions in the expression of mRNA and protein of these genes (Fig. 4A–D). Similar downregulation of PCNA and ORC6 protein and mRNA was observed in HP518-treated MFM-223 cells (Fig. S8A, B). Under conditions of androgen deprivation or supplementation with R1881, the expression of these genes remained unchanged, indicating that AR regulates a specific subset of DNA replication genes independently of androgen (Fig. S9A, B).

**Fig. 4:**
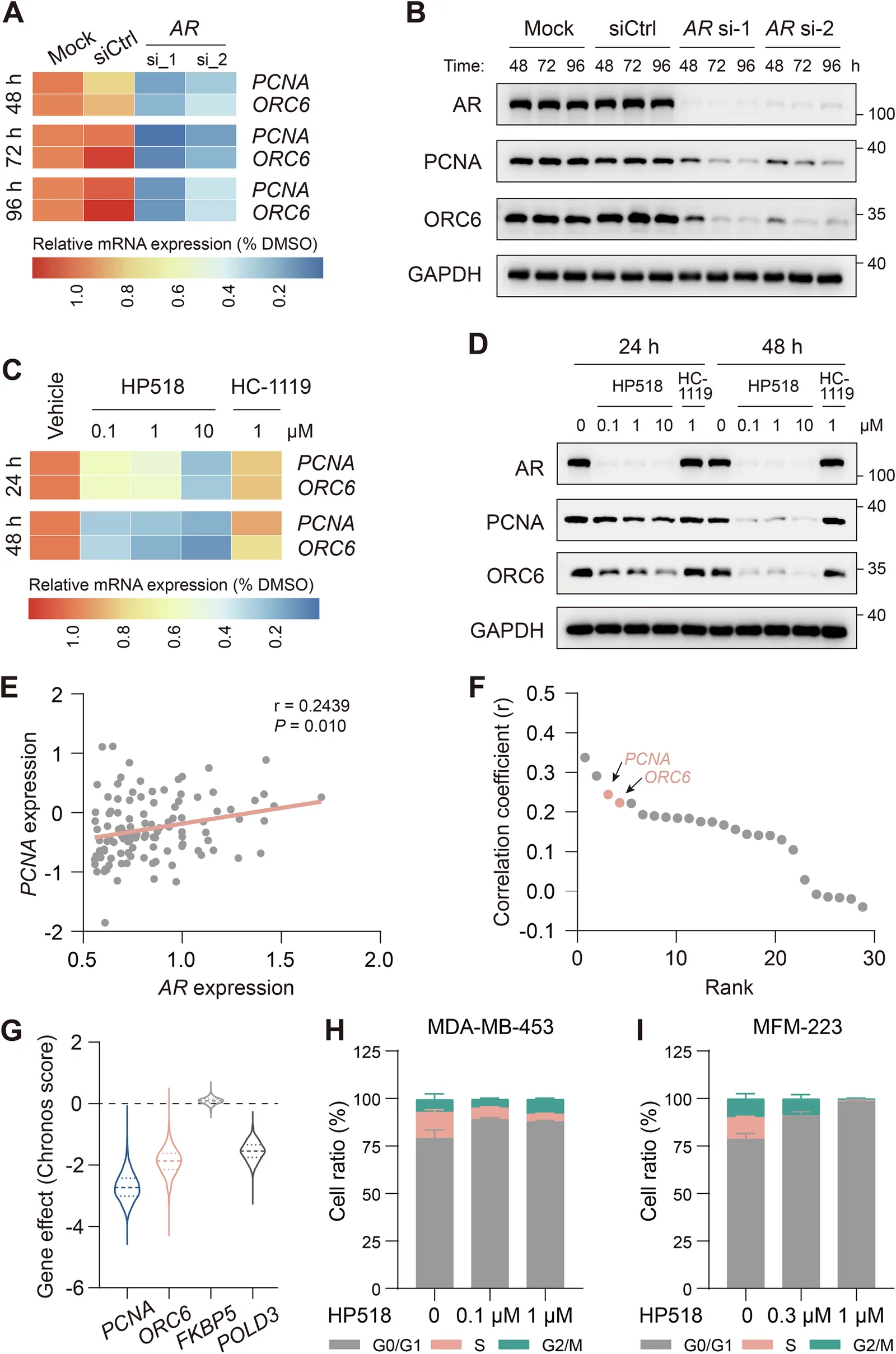
*PCNA* and *ORC6* expressions are positively correlated with AR. **A** Heat map of relative mRNA expression of *PCNA* and *ORC6* in MDA-MB-453 cells transduced with mock and siRNAs targeting control or *AR* for 48, 72, and 96 h. *n* = 3. **B** Immunoblots of AR, PCNA, and ORC6 in MDA-MB-453 cells transduced with mock and siRNAs targeting control or *AR* for 48, 72, and 96 h. **C** Heat map of relative mRNA expression of *PCNA* and *ORC6* in MDA-MB-453 cells with the indicated treatments versus vehicle for 24 and 48 h. *n* = 3. **D** Immunoblots of AR, PCNA, and ORC6 in MDA-MB-453 cells treated with HP518 and HC-1119 at the indicated concentrations for 24 and 48 h. **E** Scatterplot displaying the correlation of *PCNA* expression levels with *AR* expression levels in AR-positive TNBCs (*n* = 111). Data normalization was performed for sequencing data from different datasets. The Pearson correlation coefficient and two-tailed *P*-value were indicated. Source data are provided as a supplementary data file. **F** Rank plot displaying Pearson correlation coefficients of HP518-regulated DNA replication-related gene (Fig. 3B) expression levels with *AR* expression levels in AR-positive TNBCs (*n* = 111). *PCNA* and *ORC6* were labeled as pink dots. **G** Violin plot of gene effects (Chronos score) for *PCNA*, *ORC6*, *FKBP5*, and *POLD3* among 1100 cancer cell lines (DepMap Public 22Q1), where *FKBP5* and *POLD3* serve as the negative control and positive control, respectively. Source data are provided as a supplementary data file. Cell-cycle distribution of MDA-MB-453 (**H**) and MFM-223 (**I**) cells after treatment with DMSO or HP518 for 72 h. Quantification was performed by flow cytometry. *n* = 3.

The finding that AR promotes the expression of DNA replication genes was supported by sequencing data showing a positive correlation between *AR* expression and expression of *PCNA* and *ORC6* in 111 AR-positive TNBC patients (Fig. 4E, F, Table S2).

To determine whether the reduced expression of *PCNA* and *ORC6* contributes to HP518-induced cytotoxicity, we queried genome-wide CRISPR/Cas9 screenings in the DepMap database and found these genes to be essential in over 1000 cancer cell lines (Fig. 4G, Table S3). Consistent with this, cell-cycle analysis using EdU incorporation and flow cytometry revealed that HP518 treatment led to a dose-dependent reduction in the S-phase population, accompanied by accumulation of cells in the G0/G1 phase in AR-positive TNBC cells, indicating impaired G1/S transition (Fig. 4H, I). Together, we demonstrated that in TNBC cells, AR promotes the transcription of a subset of DNA replication genes essential for cancer cell survival, independent of androgen.

### AR‑mediated *PCNA* transcription depends on FoxA1 and GATA3

In the CUT&Tag assay, we further characterized the AR–chromatin interaction by identifying DNA motifs whose occupancy was reduced following HP518 treatment. The top depleted motifs were diverse and distinct from classical androgen response elements (AREs) (Fig. S10), suggesting that AR may not directly bind chromatin but instead function through alternative mechanisms or cofactors. On the basis of these data, we hypothesized that AR locates at the promoter region of DNA replication genes and regulates their transcription through recruitment by other coactivators, resulting in indirect interaction with DNA.

Forkhead box protein A1 (FoxA1) is a pioneer transcription factor that binds to and opens condensed chromatin through its winged-helix DNA-binding domains [14–18]. GATA binding protein 3 (GATA3) is another transcription factor that pioneers new binding sites in inaccessible chromatin, reprogramming the local chromatin structure to become accessible [19, 20]. Previous studies have shown that AR interacts with FoxA1 [21] and GATA3 [22, 23] in breast cancer cells, but the biological relevance of these interactions in TNBC remains unclear. Given that collaborating transcription factors or coactivators can facilitate nuclear receptor binding in specific regions [17, 24, 25], we investigated whether FoxA1 and GATA3 are necessary for AR-regulated transcription of DNA replication genes. Knockdown of *FoxA1* and *GATA3* using siRNAs in MDA-MB-453 cells led to robust reductions in both mRNA and protein levels of *PCNA* and *ORC6* (Fig. 5A–C), as well as decreased cell viability (Fig. S11A). Consistent with MDA-MB-453 cells, siRNA-mediated knockdown of either *FoxA1* or *GATA3* reduced cell proliferation in both MFM-223 (LAR subtype) and SUM159PT (mesenchymal stem-like, MSL subtype) cell lines (Fig. S11B, C). This suggests that FoxA1, GATA3, and AR share a common pathway critical for TNBC cell survival.

**Fig. 5:**
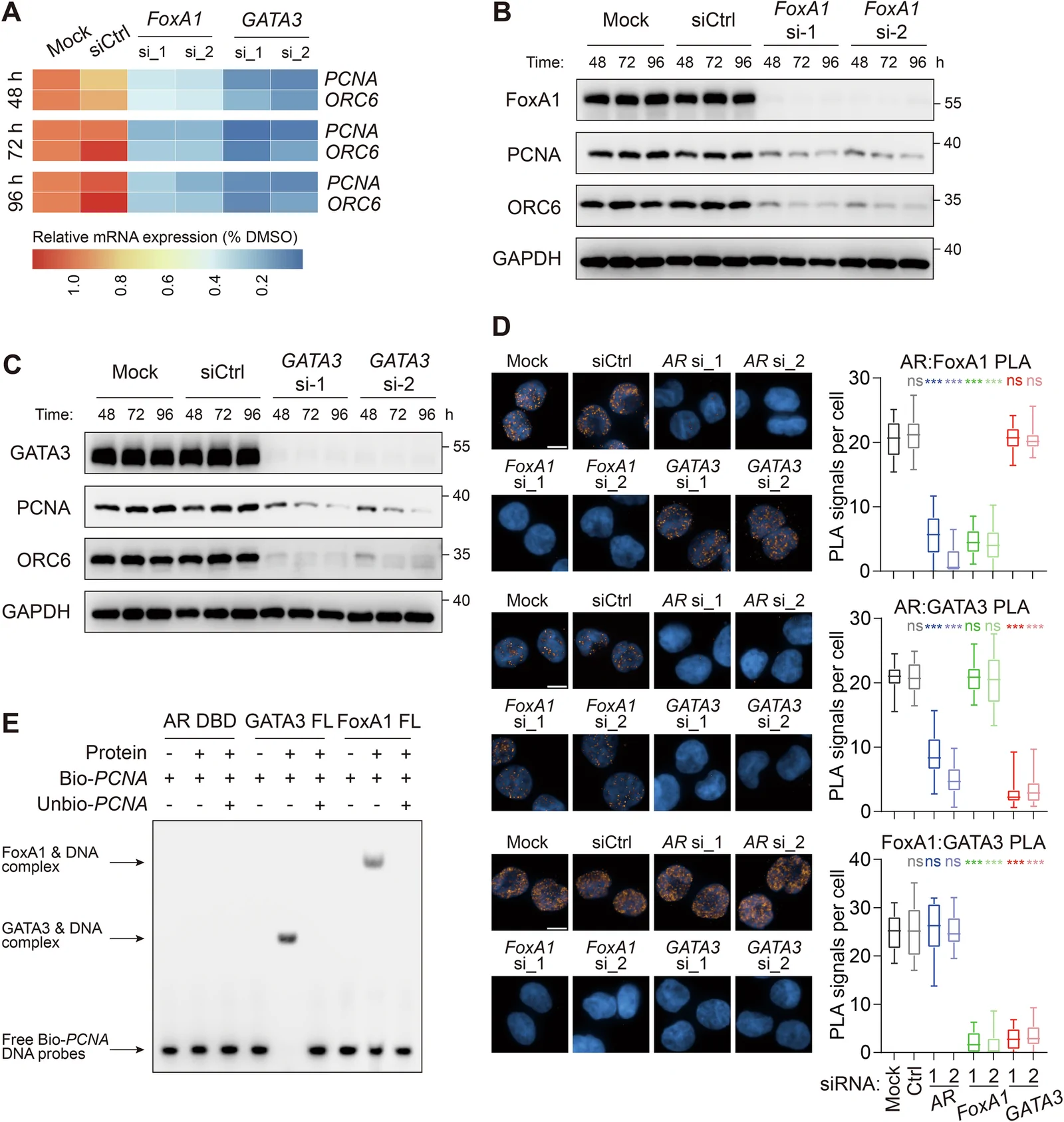
FoxA1 and GATA3 are required for AR-mediated *PCNA* and *ORC6* transcription. **A** Heat map of relative mRNA expression of *PCNA* and *ORC6* in MDA-MB-453 cells transduced with mock and siRNAs targeting control, *FoxA1*, or *GATA3* for 48, 72, and 96 h. *n* = 3. Immunoblots of AR, PCNA, and ORC6 in MDA-MB-453 cells transduced with mock and siRNAs targeting control, *FoxA1* (**B**), or *GATA3* (**C**) for 48, 72, and 96 h. **D** Representative PLA images (left) and quantification (right) of the indicated protein interactions in MDA-MB-453 cells transduced with mock, control, *AR*, *FoxA1*, or *GATA3* siRNAs for 48 h. *n* = 3. PLA signals were quantified from at least 40 different images and are shown as box and whisker plots displaying the median PLA signals per cell, with whiskers incorporating the min-to-max of all data. Significance between each group and mock was analyzed using one-way ANOVA with Dunnett’s multiple comparison tests (ns: not significant; ***P < 0.001). Scale bars, 10 μm. **E** EMSA analysis of the binding between the biotinylated-*PCNA* probe (Bio-*PCNA*) and recombinant AR DNA-binding domain (AR-DBD), full-length FoxA1 (FoxA1 FL), and full-length GATA3 (GATA3 FL) proteins. The Bio-*PCNA* probe was generated from the CUT&Tag tracks at the promoter region, and the unbiotinylated probe (Unbio-*PCNA*) served as a competitor. The binding was analyzed by immunoblotting with a biotin antibody.

We then examined whether AR, FoxA1, and GATA3 interact and form a complex in MDA-MB-453 cells using proximity ligation assays (PLA). The AR:FoxA1 and AR:GATA3 PLAs generated fluorescent puncta in the nucleus (Fig. 5D). These puncta were reduced following targeted degradation of AR using HP518 and AR knockdown. Knockdown of *FoxA1* specifically disrupted the AR:FoxA1 interaction but not AR:GATA3, while knockdown of *GATA3* only disrupted the AR:GATA3 interaction but not AR:FoxA1 (Fig. 5D, Fig. S12A, B). Similarly, the FoxA1:GATA3 PLA produced fluorescent puncta in the nucleus, which decreased with *FoxA1* and *GATA3* knockdown, but were unaffected by HP518 treatment or *AR* knockdown (Fig. 5D, Fig. S12C). These results demonstrate that AR, FoxA1, and GATA3 can interact independently of one another.

To further investigate whether AR, FoxA1, and GATA3 interact with the target gene’s promoter, we conducted electrophoretic mobility shift assays (EMSA) using purified recombinant AR DNA-binding domain (AR-DBD), full-length FoxA1 (FoxA1 FL), and full-length GATA3 (GATA3 FL). We first validated their activity by confirming complex formation with oligos containing their classical binding motifs (Fig. S13A–D). Using a synthetic DNA probe from CUT&Tag peaks at the *PCNA* promoter, we found that FoxA1 and GATA3 formed protein-DNA complexes, while AR-DBD did not, supporting the hypothesis that AR does not directly interact with the target DNA (Fig. 5E).

Together, these findings support a model in which AR is recruited to DNA replication gene promoters through FoxA1 and GATA3, forming an AR–FoxA1–GATA3 regulatory axis that drives TNBC cell proliferation.

### HP518 inhibits tumor growth in AR-positive TNBC patient-derived xenografts

We previously demonstrated that AR depletion inhibits TNBC cell growth in *vitro*. To assess the translational potential of AR degradation, we established two AR‑positive patient‑derived xenograft (PDX) models (Fig. S5 and S14A). In the BR5009 model, HP518 induced robust, dose‑dependent tumor suppression (Fig. 6A). In contrast, ARV‑110 showed weaker anti-tumor activity at an equivalent dose, consistent with its lower AR‑degrading potency in tumors (Fig. 6A, B). *PIK3CA* gain-of-function mutations are frequently observed in AR-positive TNBC patients [26]. Genetic profiling of this patient revealed the C420R mutation in the *PIK3CA* gene. Notably, HP518 combined with the PI3Kα inhibitor alpelisib [27, 28] exhibited enhanced efficacy in this PDX model (Fig. 6A). In a second, chemo‑naïve PDX model (LD1‑2009‑361825), HP518 potently degraded AR and, in combination with paclitaxel, markedly inhibited tumor growth (Fig. 6C, D). When paclitaxel dosing stopped, HP518 alone maintained the combination’s efficacy while the tumors in the paclitaxel group grew back (Fig. 6C). Consistently, in matched ex vivo models derived from the same BR5009 and LD1-2009-361825 tumors, HP518 in combination with alpelisib or paclitaxel demonstrated enhanced anti-proliferative activity (Fig. S14B), in line with the in vivo observations.

**Fig. 6:**
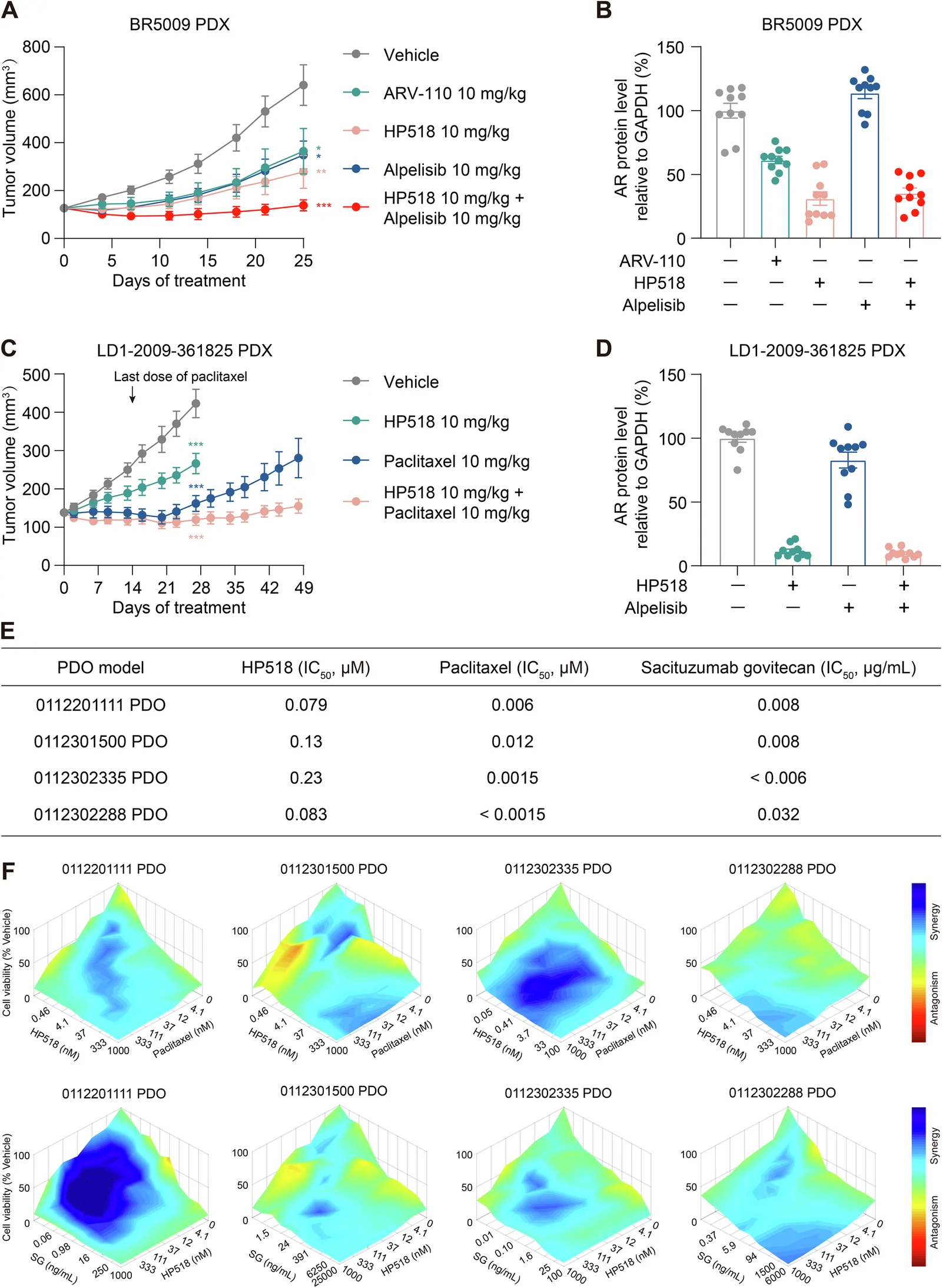
HP518 inhibits tumor progression in AR-positive TNBC patient-derived xenografts. **A** Tumor growth analysis of AR-positive TNBC patient (No. BR5009)-derived xenografts in NOD/SCID mice treated with the indicated. ARV-110, HP518, and alpelisib were administered orally once daily. Significance between each group versus vehicle was analyzed using one-way ANOVA with Dunnett’s multiple comparison tests (**P < 0.01, ***P < 0.001). Data expressed as mean ± standard error of the mean (SEM) (*n* = 10). **B** Quantifications of immunoblots of AR in BR5009 PDX tumor samples. Each circle represents an individual sample. Data expressed as mean ± SEM (*n* = 10). **C** Tumor growth analysis of AR-positive TNBC patient (No. LD1-2009-361825)-derived xenografts in CB-17 SCID mice treated with the indicated. HP518 and paclitaxel were administered orally once daily and intravenously once weekly, respectively. Paclitaxel was administered on Days 0, 7, and 14, with treatment discontinued thereafter in both the paclitaxel and paclitaxel + HP518 groups, and tumor regrowth was significantly delayed in the combination therapy group compared to the paclitaxel monotherapy group. Significance between each group versus vehicle was analyzed using one-way ANOVA with Dunnett’s multiple comparison tests (**P < 0.01, ***P < 0.001). Data expressed as mean ± SEM (*n* = 10). **D** Quantifications of immunoblots of AR in LD1-2009-361825 PDX tumor samples. Each circle represents an individual sample. Data expressed as mean ± SEM (*n* = 10). **E** Anti-proliferative potency of HP518, paclitaxel, and sacituzumab govitecan in four AR-positive TNBC patient-derived organoid (PDO) models after 7 days of treatment. *n* = 2. **F** Combinatorial efficacy and synergy analysis of HP518 with paclitaxel or sacituzumab govitecan. HP518 was combined with either paclitaxel (upper panels) or sacituzumab govitecan (lower panels) in four AR-positive TNBC PDO models for 7 days of treatment. The combination effects were evaluated using the Highest Single Agent (HSA) model in Combenefit. Synergy scores were calculated by comparing observed responses with the expected effects defined as the maximal response of either single agent and visualized as a synergy map projected onto the dose–response (D–R) surface. *n* = 3.

To further account for inter-patient heterogeneity, we expanded our analysis to four additional independent 3D patient-derived organoid (PDO) models, all of which formed well-defined tumor spheroids (Fig. S15). HP518 demonstrated robust, dose-dependent anti-proliferative activity, with IC_50_ values of 0.079 μM, 0.13 μM, 0.23 μM, and 0.083 μM, respectively (Fig. 6E, Fig. S16). Given that cytotoxic chemotherapy remains the standard of care for TNBC [29], and that TROP2-targeted antibody–drug conjugates (ADCs)—such as sacituzumab govitecan (SG), a first-in-class TROP2 ADC with broad clinical adoption—are widely used following chemotherapy failure [30], we further evaluated combination strategies in these PDO models. Specifically, HP518 was combined with paclitaxel as well as SG across all four PDOs. Notably, consistent synergistic anti-tumor activity was observed across all models (Fig. 6F), which may be attributed to HP518-induced impairment of DNA replication and repair, sensitizing TNBC cells to both mitotic stress and replication-associated DNA damage.

Together, these findings demonstrate that HP518 exhibits consistent and robust anti-tumor activity across 2D, 3D, and in vivo tumor models, both as a monotherapy and in combination settings, supporting its potential as a versatile therapeutic strategy in AR-positive TNBC, including use with PI3Kα inhibitors, chemotherapy, and ADC-based therapies across both frontline and post-chemotherapy treatment settings.

## Discussion

Inhibitors targeting AR signaling have revolutionized prostate cancer treatment, especially for castration-resistant cases [31–34]. However, the role of AR in breast cancer remains complex and contradictory, particularly regarding ER status [35]. Previous studies focused on traditional AR pathways dependent on ligand binding, leading to the use of AR inhibitors for treating ER-negative breast cancer. Despite this, anti-androgen therapies like enzalutamide, bicalutamide, and abiraterone have shown limited anti-tumor efficacy in clinical trials. Zhao et al. reported that the AR PROTAC ARD-61 inhibited MDA-MB-453 growth both in vitro and in vivo, but did not explore the mechanisms or differences between PROTACs and AR antagonists in TNBC cells [36]. Our study presents novel genetic, biochemical, and functional evidence that AR acts as a pro-survival signaling hub in AR-positive TNBC cells, regulating DNA replication genes through FoxA1 and GATA3, independently of androgen signaling. Notably, while PCNA and ORC6 were used as representative AR-regulated targets, our transcriptomic analyses indicate a broader suppression of DNA replication and DNA damage repair pathways, suggesting that AR degradation induces a coordinated disruption of replication machinery rather than a single-gene effect. This mechanism contrasts with prostate cancer, underscoring the potential of targeted AR degradation by HP518 as a promising therapeutic strategy for TNBC (Fig. 7).

**Fig. 7:**
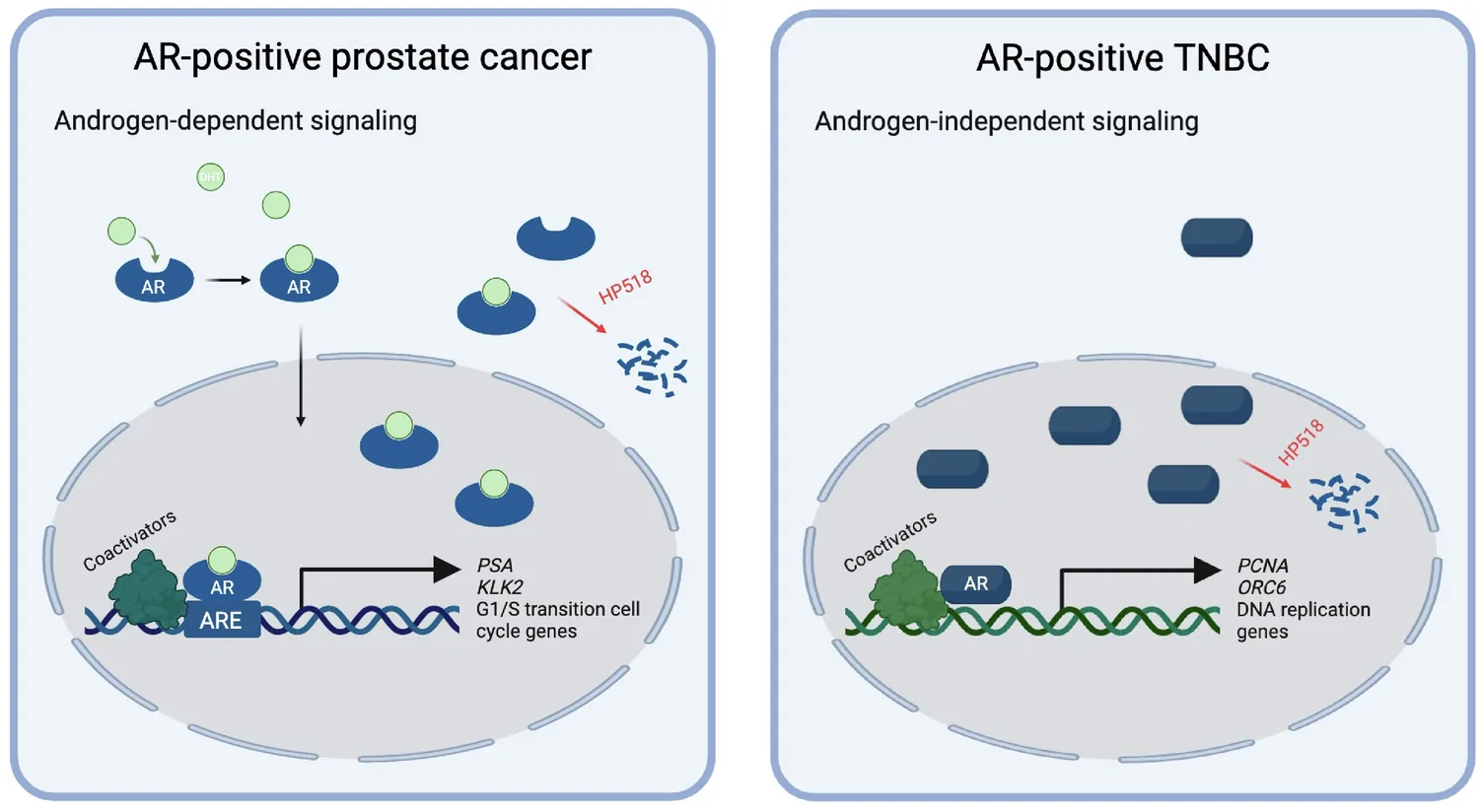
Model of the different roles of AR in prostate cancer and TNBC. Schematic illustration depicting the different roles of AR in prostate cancer and TNBC, and the underlying mechanism of action of HP518 as an AR PROTAC degrader in suppressing tumor growth. Left panel: canonical androgen-dependent AR signaling in prostate cancer: upon binding to androgen ligands, AR undergoes a conformational change and translocates from the cytoplasm to the nucleus, where AR binds to AREs within the promoter regions of target genes. This binding recruits co-regulators and the transcriptional machinery, ultimately driving the expression of downstream genes involved in cellular growth, differentiation, and survival, such as *PSA*, *KLK2*, and G1/S transition cell-cycle genes. Right panel: ligand-independent role of AR in AR-positive TNBC cells: AR is located in the nucleus and binds to distinct co-regulators, such as GATA3 and FoxA1, driving the expression of DNA replication-related genes, including *PCNA* and *ORC6*, which are different from those in prostate cancer cells. HP518 inhibits AR signaling and cell proliferation in prostate cells and AR-positive TNBC cells via AR degradation.

GATA3, a key transcription factor in breast cancer, regulates ER expression [37, 38] and strongly correlates with AR presence in TNBC patients [39]. Although GATA3-AR interaction has been reported, its biological significance in TNBC remains unclear [22]. FoxA1, a “pioneer factor,” also regulates AR function in both breast and prostate cancers, with its expression correlating with AR in TNBC, identifying a subgroup of patients with poor prognosis [40]. While GATA3 acts upstream of FoxA1 in ER-positive breast cancer, FoxA1 facilitates AR binding to chromatin in prostate cancer [16, 20, 25]. However, the exact roles of AR, GATA3, and FoxA1 in TNBC remain unclear. Our study is the first to identify the AR–FoxA1–GATA3 regulatory axis in this context, suggesting that FoxA1 or GATA3 may regulate AR-dependent gene targets in TNBC.

Notably, while AR signaling in prostate cancer has been extensively characterized as a canonical androgen-driven transcriptional program, the present study is focused on delineating the TNBC-specific AR regulatory landscape. Accordingly, we did not perform a systematic side-by-side mechanistic reconstruction of AR signaling in prostate cancer models. Instead, our conclusions are supported by internal comparisons within TNBC systems, where canonical AR target genes are not responsive to either AR degradation or antagonism (Fig. S6), highlighting a lineage-specific divergence in AR function.

HP518, developed by Hinova Pharmaceuticals, specifically degrades AR with a DC_50_ < 1 nM and inhibits tumor growth in both prostate cancer and TNBC models. Our results demonstrate HP518’s efficacy in AR-positive TNBC PDX models and its potential synergistic effects when combined with a PI3Kα inhibitor and chemotherapy. A Phase Ia study in Australia showed promising results for mCRPC patients (NCT05252364), and a Phase I/II trial is ongoing in China for mCRPC (NCT06155084). HP518 has outperformed other AR PROTACs in TNBC studies (Figs. 2B–F and 6A), indicating its potential as a leading AR-targeted therapy. Recently, the FDA granted Fast Track designation for HP518 in treating AR-positive TNBC. Further clinical trials are needed to confirm its efficacy as a monotherapy or in combination therapies. AR is also frequently expressed in other cancers including primary hepatocyte cancer, gastric cancer, bladder cancer, and pancreatic cancer [41, 42], suggesting that HP518 may extend to the treatment of these cancers.

## Methods

### Cell culture

MDA-MB-453, SUM159PT, and HCC1937 cells were purchased from BNCC. MFM-223 and MDA-MB-468 cells were purchased from COBIOER. All cell lines were mycoplasma-negative and authenticated by STR profiling. MDA-MB-453, MDA-MB-468, SUM159PT and HCC1937 cells were maintained in RPMI 1640 (Hyclone) with 10% fetal bovine serum (FBS, Biolnd) and 1% penicillin-streptomycin (Gibco), except for MFM-223 in minimum essential medium (Hyclone) with 10% fetal bovine serum, 1% non-essential amino acids (Gibco), and 1% penicillin-streptomycin, at 37 °C and 5% CO_2_. For androgen deprivation, cells were cultured in medium with 10% charcoal-stripped FBS (CSS, Biolnd) and 1% penicillin-streptomycin for 3 days.

### Compounds

MG132 (HY-13259), MLN7243 (HY-100487), MLN4924 (HY-70062), lenalidomide (HY-A0003), and enzalutamide (HY-70002) were purchased from MedChemExpress (MCE). HP518 and HC-1119 were synthesized in-house.

### Antibodies

The following antibodies were used in this study: anti- AR (Cell Signaling Technology, 5153S for Western blot and proximity ligation assay), anti- AR (Abcam, ab9474 for proximity ligation assay), anti- AR (Invitrogen, PA1-9005 for immunofluorescence staining), anti- Na^+^K^+^ ATPase (Abcam, ab76020), anti- PCNA (Abclonal, A12427), anti- ORC6 (Invitrogen, PA5-113534), anti- FoxA1 (Cell Signaling Technology, 53528S), anti- GATA3 (Cell Signaling Technology, 5852S), anti- Lamin B1 (Cell Signaling Technology, 13435), and anti- GAPDH (Cell Signaling Technology, 2118L).

### In vitro cell growth assay

For the androgen-independent cell growth assay, cells were supplemented with 0, 1 nM, 10 nM, or 100 nM R1881 (Macklin), after being cultured in androgen-deprived medium for 3 days. For the siRNA-transduced cell growth assay, cells were transduced with mock, control siRNA, or siRNAs targeting *AR*, *FoxA1*, or *GATA3*. The cell growth marked by cell viability was quantified every 2–3 days using a CellTiter-Glo luminescent cell viability assay kit (Promega Corporation) and FLUOstar Omega microplate reader (BMG Labtech).

### Colony formation assay

For siRNA transfection, MDA-MB-453 (8 × 10^5^ cells/well) and MFM-223 (6 × 10^5^ cells/well) cells were seeded in 6-well plates and transfected with mock or 30 pmol of either control or *AR*-targeting siRNAs using 9 μL Lipofectamine™ RNAiMAX (Invitrogen). After 48 h transfection, cells were harvested and re-seeded into 6-well plates at densities of 3000 (MDA-MB-453) and 600 (MFM-223) cells per well. After a 14-day incubation period, cells were fixed and visualized via crystal violet staining.

### Nuclear and cytoplasmic fractionation

MDA-MB-453 (8 × 10^5^ cells/well) and MFM-223 (5 × 10^5^ cells/well) cells were seeded in 6-well plates. For androgen deprivation, cells were cultured in medium supplemented with charcoal-stripped fetal bovine serum (CSS) or normal FBS for 72 h. Subsequently, cells were treated with 100 nM R1881 (a synthetic androgen) or DMSO (vehicle control) for 24 h prior to protein extraction. To evaluate the subcellular distribution of AR, cytoplasmic and nuclear fractions were isolated using the NE-PER Nuclear and Cytoplasmic Extraction Reagents (Thermo Fisher Scientific). Briefly, cell pellets were resuspended in ice-cold Cytoplasmic Extraction Reagent I (CER I) and incubated on ice for 10 min, followed by the addition of CER II. After centrifugation, the supernatant (cytoplasmic fraction) was collected. The remaining nuclear pellet was suspended in ice-cold Nuclear Extraction Reagent (NER) for 40 min at 4 °C with periodic vortexing. Protein concentrations of both fractions were determined by BCA assay (Beyotime).

### Immunoblots for whole-protein lysates

Standard SDS-PAGE and Western blotting procedures were used with the following modifications. For preparation of total lysates, cells were rinsed with DPBS to remove residual medium and then lysed in 20 mM HEPES-NaOH pH 8.0, 10 mM NaCl, 2 mM MgCl_2_, 1% SDS freshly supplemented with 1 unit/mL of benzonase (Yeasen) and 1x protease inhibitor cocktail (Absin) for 10 min at room temperature. Protein concentrations of the lysates were quantified using a BCA protein assay kit (Beyotime). For blotting proteins, an equal amount of lysate was run on SDS-PAGE protein gels and then transferred to polyvinylidene fluoride (PVDF) membranes with a pore size of 0.2 μm (Millipore). Membranes were blocked in 5% nonfat milk Tris-buffered saline with Tween 20 (TBST) and incubated with primary antibodies overnight at 4 °C. The membranes were then washed in TBST, incubated with secondary antibodies for 1 h at room temperature, and washed three times in TBST before Western blot detection on an imager system (Tanon).

### Cell viability assay

Human TNBC cell lines (MDA-MB-453, MFM-223, MDA-MB-468, and HCC1937), primary cells isolated from AR-positive TNBC patient samples (BR5009, BR9464, and LD1-2009-361825) and AR-positive TNBC PDOs (0112201111, 0112301500, 0112302335, and 0112302288) were utilized in this study. For cell viability assays, cells or PDOs were seeded into 96-well microplates (Corning) at an optimized density to ensure logarithmic growth throughout the treatment period. Cells were treated with serial dilutions of tested compounds for 5 or 7 days. Cell viability was measured using a CellTiter-Glo luminescent cell viability assay kit (Promega Corporation). Luminescence was recorded by a FLUOstar Omega microplate reader (BMG Labtech). The half-maximal inhibitory concentration (IC_50_) was determined with GraphPad Prism using baseline correction (by normalizing to vehicle (dimethyl sulfoxide, DMSO) control), the asymmetric (five parameter) equation, and least squares fit.

### Immunofluorescence (IF) staining

MDA-MB-453 and MFM-223 cells cultured in medium supplemented with FBS or charcoal-stripped FBS (CSS) were treated with vehicle or 100 nM R1881 for 24 h and then fixed with 4% paraformaldehyde (Beyotime) for 15 min at room temperature. For IF microscopy, cells were blocked in 5% goat serum solved in PBS containing 0.1% Triton-X100 for 1 h and incubated with primary antibodies (anti-AR, Invitrogen, PA1-9005, 1:200; anti-Na^+^K^+^ ATPase, Abcam, ab76020, 1:200) diluted in PBS containing 0.1% Triton-X100 overnight at 4 °C and then incubated with secondary antibodies (donkey anti-Goat IgG (H+L) Alexa Fluor 488, Invitrogen, A11055, 1:1000; donkey anti-Rabbit IgG (H+L) Alexa Fluor 555, Invitrogen, A31572, 1:1000) for 1 h at room temperature, protecting from light. Nuclei were counterstained with 4′,6-diamidino-2-phenylindole (DAPI) before mounting. Fluorescence cells were scanned, imaged, and quantified using an Operetta CLS high-content analysis system.

### RNA-seq and gene ontology analysis

Total cellular RNA was purified from cells treated with DMSO (vehicle), 100 nM HP518, or 1 μM HC-1119 for 24 h. Standard RNA-seq was performed by Sangon Biotech. Sequencing reads were aligned to the human hg38 reference transcriptome using Bowtie2 [43] followed by gene-level quantification with RSEM [44]. Differential gene expression analyses were performed with DESeq2 [45] with the following cutoff: absolute log2-transformed fold change greater than 1 or less than −1 and *P*-value less than 0.01. Gene ontology analysis of differentially expressed genes was performed with clusterProfiler [46].

### Quantitative PCR (qPCR)

Total cellular RNA was extracted from cells using TRIZOL (Thermo Fisher). RNA was converted into cDNA using SuperScript™ III first-strand synthesis supermix (Thermo Fisher) according to the manufacturer’s instructions. Quantitative PCR was performed using Platinum™ SYBR™ green qPCR supermix (Thermo Fisher) in a CFX96 real-time system (Bio-Rad). The primers used were as follows: *PCNA*, forward 5′-cggagtgaaattttctgcaagtggagaac-3′, reverse 5′-gtgtcaccgttgaagagagtggag-3′; *ORC6*, forward 5′-agagaagattggacagcaggtcgac-3′, reverse 5′-gtggtttatgtggcatctcctctacc-3′; *SLC2A1*, forward 5′-ttgcaggcttctccaactggac-3′, reverse 5′-cagaaccaggagcacagtgaagatg-3′; *FKBP5*, forward 5′-gcgaaggagaagaccacgacat-3′, reverse 5′-taggcttccctgcctctccaaa-3′; *NDRG1*, forward 5′-atcacccagcactttgccgtct-3′, reverse 5′-ggactccaggaagcatttcagcca-3′; *CAMKK2*, forward 5′-tcaaaccactctctgaagaccaggc-3′, reverse 5′-ttctccgaccaggaggttggaag-3′; *UBE2C*, forward 5′-ctggcgataaagggatttctgcc-3′, reverse 5′-gcgagagcttatacctcaggtcttca-3′; *CDC20*, forward 5′-cggaagacctgccgttacattc-3′, reverse 5′-accagagcttgcactccacaggta-3′; *CDK1*, forward 5′-ggaaaccaggaagcctagcatc-3′, reverse 5′-ggatgattcagtgccattttgccag-3′; *ANAPC10*, forward 5′-cgggaaattgggtcacaagctg-3′, reverse 5′-ggatgttcactaaatgaggctgggaac-3′; *TBP* (Sangon, B661108). All quantitative PCR programs were 95 °C for 3 min, 40 cycles of 95 °C for 10 s and 60 °C for 30 s, followed by a melting curve. Relative mRNA expression was evaluated after normalization for *TBP* expression using the standard ΔΔCT method.

### Bioinformatic analysis

DepMap Public 22Q1, including gene effect (Chronos), gene expression (RNA-seq), and cell lines information, was downloaded from the DepMap data portal. Chronos scores for over 1,000 cancer cell lines were extracted. Violin plots for *PCNA*, *ORC6*, *FKBP5,* and *POLD3* were generated by GraphPad Prism using default parameters.

Sequencing data of TNBC patients were downloaded from NODE (OEP000155) and NCBI (GSE154524 and GSE163882). Data normalization was performed using an integrative multi-omic analysis [47], and the *AR* score greater than 0.55 was considered AR-positive. Correlation analyses were generated in GraphPad Prism.

### Cleavage under targets and tagmentation (CUT&Tag)

AR CUT&Tag was performed using an anti-AR antibody (Cell Signaling Technology, 5153S; 1:100) by DIATRE. In brief, a total of 3 × 10^5^ MDA-MB-453 cells were treated with DMSO (vehicle), 100 nM HP518, or 1 μM HC-1119 for 8 h. After treatment, cell nuclei were isolated and incubated with the primary and secondary antibodies. The unbound antibodies were removed with Dig-wash buffer, and the pA-Tn5 adapter complex was prepared and added to the nuclei with gentle vortexing. Washed nuclei were resuspended in Tagmentation buffer, and STOP buffer was used to stop tagmentation. The library DNA was purified and amplified for sequencing using the Illumina NovaSeq 6000. Raw sequencing reads were initially processed by FastQC for quality control, then adapter sequences and poor-quality reads were removed. Clean reads were aligned to the human reference genome (hg38) using Bowtie2 [43]. SAM files were converted to BAM format using Samtools [48]. Peaks were called by MACS2 [49] with an initial threshold *P*-value of 0.01 as a cutoff. Bigwig files were generated by deeptools [50] and visualized by IGV [51]. Motif analysis was further identified with the HOMER package [52] with default parameters.

### Cell-cycle analysis

MDA-MB-453 and MFM-223 cells were seeded into 24-well plates at a cell density of 1.5 × 10^5^ cells/well and subjected to serum starvation overnight to achieve cell-cycle synchronization. Subsequently, cells were treated with the indicated concentrations of HP518 for 72 h. Cell-cycle distribution and DNA synthesis were quantified using the Click-iT™ EdU Alexa Fluor™ 647 Flow Cytometry Assay Kit (Thermo Fisher Scientific) in accordance with the manufacturer’s instructions. For each sample, a minimum of 10,000 gated single-cell events were acquired using a flow cytometer. All experiments were performed in triplicate to ensure reproducibility.

### Proximity ligation assay (PLA)

MDA-MB-453 cells were treated with DMSO (vehicle), 100 nM HP518, or 1 μM HC-1119 for 8 h or transduced with mock, control siRNA, or siRNAs targeting *AR*, *FoxA1*, or *GATA3* for 48 h. After treatment, cells were fixed with 4% paraformaldehyde (Beyotime) for 15 min at room temperature, and the proximity of the interested proteins was visualized using Duolink In Situ Detection reagents (Sigma) according to the manufacturer’s instructions. Briefly, fixed cells were blocked with blocking buffer for 1 h at room temperature and labeled with antibodies targeting the indicated proteins overnight at 4 °C. After a washing step, PLA probes were added and incubated for 1 h at 37 °C. Cells were subsequently washed and incubated with ligase for 30 min at 37 °C, followed by oligonucleotide amplification with DNA polymerase for 100 min at 37 °C. Nuclei were counterstained with DAPI before mounting and cells were imaged by an Operetta CLS high-content analysis system (PerkinElmer).

### Electrophoretic mobility shift assay (EMSA)

Recombinant AR DNA-binding domain (amino acid 557 - 644) protein was purified for SF9 insect cells by Viva Biotech. Recombinant full-length FoxA1 and GATA3 proteins were purchased from ORIGENE. The (un) biotinylated DNA probes generated from the CUT&Tag peak at the promoter of the *PCNA* gene (Sense 5′-CTAGAAAGACAACGACCACTCTGCTACGCCTGCAACCGTT-3′; antisense 5′-AACGGTTGCAGGCGTAGCAGAGTGGTCGTTGTCTTTCTAG-3′) were synthesized by Sangon Biotech. Briefly, 5 μM AR-DBD, 1.3 μM full-length FoxA1, or 1 μM full-length GATA3 was incubated with 1 nM biotinylated-*PCNA* probe in 20 μL of reaction binding buffer (20 mM Tris pH 7.4, 20 mM NaCl, 2 mM MgCl_2_) for 1 h at 25 °C. For competitive binding, 200 nM unbiotinylated-*PCNA* probe was added as a competitor. After incubation, 5 μL of 5× loading buffer was added, and 20 μL per sample was loaded onto 6% DNA retardation gels (WSHTBio) equilibrated in 0.5× TBE buffer. Electrophoresis was performed at 100 V for 30–60 min and signals were detected by a LightShift chemiluminescent EMSA kit (Thermo Fisher).

### Mice and tumor xenograft

The animal studies were approved by the Institutional Animal Care and Use Committee of CrownBio and LIDE prior to execution. During the studies, the care and use of animals were conducted in accordance with the regulations of the Association for Assessment and Accreditation of Laboratory Animal Care (AAALAC). Patient-derived tumor tissues used for PDX studies were obtained with informed consent in accordance with applicable ethical and regulatory requirements.

For all xenograft studies, mice were randomized into treatment groups at treatment initiation using a stratified randomization approach based on tumor volume to ensure comparable mean tumor volumes across groups.

AR-positive TNBC patient BR5009-derived xenografts were performed by CrownBio. Female NOD/SCID mice were obtained from GemPharmatech and used for the xenograft experiments between 6 and 8 weeks of age. BR5009 PDXs were harvested from tumor-bearing mice, processed into 2–3 mm^3^ fragments, and implanted subcutaneously in the right flanks of NOD/SCID mice. Treatments were initiated when the average tumor volume reached 126 mm^3^. HP518 and ARV-110 were prepared in 5% DMSO, 15% HS-15, and 80% D5W in DI water and administered orally once per day (3 mg/kg/day, 10 mg/kg/day, or 30 mg/kg/day). Alpelisib (MCE, HY-15244) was prepared in sterilized ddH_2_O with 30% HP-β-CD and administered orally once per day (10 mg/kg/day). For the HP518/alpelisib combination, drugs were given at the same concentration and schedule as single agents. Tumor growth was monitored twice per week by measuring tumor size in two orthogonal dimensions, and tumor volume was calculated by the following equation: tumor volume = (length × width^2^)/2. Before the last dosing, mice were starved for 12 h. Plasma and tumor samples were collected at 4 h and 16 h post-the-last dosing to further analyze drug exposure and AR protein expression by LC-MS/MS and Western blotting, respectively.

AR-positive TNBC patient LD1-2009-361825-derived xenografts were performed by LIDE. Female CB-17 SCID mice were obtained from Charles River and used for the xenograft experiments between 6 and 9 weeks of age. LD1 PDXs were harvested from tumor-bearing mice, processed into 3 mm^3^ fragments, and implanted subcutaneously in the right flanks of CB-17 SCID mice. Treatments were initiated when the average tumor volume reached 138 mm^3^. HP518 was prepared in 5% DMSO, 15% HS-15, and 80% D5W in DI water and administered orally once per day (10 mg/kg/day). Paclitaxel (MCE, HY-B0015) was prepared in sterilized ddH_2_O with 5% DMSO and 5% Tween 80 and administered intravenously once per week (10 mg/kg/week). For HP518/paclitaxel combination, drugs were given at the same concentration and schedule as single agents. Tumor growth was monitored twice per week by measuring tumor size in two orthogonal dimensions, and tumor volume was calculated by the following equation: tumor volume = (length × width^2^)/2. Before the last dosing, mice were starved for 12 h. Tumor samples were collected at 4 h and 16 h post-the-last dosing to further analyze AR protein expression by Western blotting.

### siRNA sequences used for functional assays

Control siRNA (TSGXS113) was purchased from Tsingke. *AR* si_1, sense 5′-ccugcuaaucaagucacacautt-3′, antisense 5′-augugugacuugauuagcaggtt-3′; *AR* si_2, sense 5′-caccaaugucaacuccaggautt-3′, antisense 5′-auccuggaguugacauuggugtt-3′; *FoxA1* si_1, sense 5′-cuacucguacaucucgcucautt-3′, antisense 5′-augagcgagauguacgaguagtt-3′; *FoxA1* si_2, sense 5′-gcguacuaccaagguguguautt-3′, antisense 5′-auacacaccuugguaguacgctt-3′; *GATA3* si_1, sense 5′-ccucugcuucauggaucccuatt-3′, antisense 5′-uagggauccaugaagcagaggtt-3′; *GATA3* si_2, sense 5′-cgagaaagagugccucaaguatt-3′, antisense 5′-uacuugaggcacucuuucucgtt-3′.

### Statistical significance

Statistical analyses were performed with GraphPad Prism. Student’s *t-*test was used to evaluate the statistically significant difference between the two sample groups. When comparing more than two independent groups, ANOVA was used to evaluate statistical significance. Multiple comparison tests were performed when ANOVA was significant. All tests were two-tailed, and *P* < 0.05 was considered statistically significant.

### Reporting summary

Further information on research design is available in the Nature Research Reporting Summary linked to this article.

## Supplementary information

Table S1

Table S2

Table S3

Supplementary material

Uncropped Western blot images

Reporting Summary

## Data Availability

The datasets generated from this study are included in the supplementary materials. Any additional information required to reanalyze the data reported in this study is available from the corresponding author upon reasonable request.

## References

[1] Zagami P, Carey LA. Triple negative breast cancer: Pitfalls and progress. npj Breast Cancer. 2022;8:95. 10.1038/s41523-022-00468-0 PMC9392735 35987766

[2] Foulkes WD, Smith IE, Reis-Filho JS. Triple-negative breast cancer. N Engl J Med. 2010;363:1938–48. 10.1056/NEJMra1001389 21067385

[3] Lehmann BD, Bauer JA, Chen X, Sanders ME, Chakravarthy AB, Shyr Y, et al. Identification of human triple-negative breast cancer subtypes and preclinical models for selection of targeted therapies. J Clin Invest. 2011;121:2750–67. 10.1172/JCI45014 PMC3127435 21633166

[4] Astvatsaturyan K, Yue Y, Walts AE, Bose S. Androgen receptor positive triple negative breast cancer: clinicopathologic, prognostic, and predictive features. PLoS ONE. 2018;13:e0197827. 10.1371/journal.pone.0197827 PMC5993259 29883487

[5] Gucalp A, Tolaney S, Isakoff SJ, Ingle JN, Liu MC, Carey LA, et al. Phase II trial of bicalutamide in patients with androgen receptor-positive, estrogen receptor-negative metastatic breast cancer. Clin Cancer Res. 2013;19:5505–12. 10.1158/1078-0432.CCR-12-3327 PMC4086643 23965901

[6] Traina TA, Miller K, Yardley DA, Eakle J, Schwartzberg LS, O’Shaughnessy J, et al. Enzalutamide for the treatment of androgen receptor-expressing triple-negative breast cancer. J Clin Oncol. 2018;36:884–90. 10.1200/JCO.2016.71.3495 PMC5858523 29373071

[7] Sakamoto KM, Kim KB, Kumagai A, Mercurio F, Crews CM, Deshaies RJ. Protacs: chimeric molecules that target proteins to the Skp1-Cullin-F box complex for ubiquitination and degradation. Proc Natl Acad Sci USA. 2001;98:8554–9. 10.1073/pnas.141230798 PMC37474 11438690

[8] Bekes M, Langley DR, Crews CM. PROTAC targeted protein degraders: the past is prologue. Nat Rev Drug Discov. 2022;21:181–200. 10.1038/s41573-021-00371-6 PMC8765495 35042991

[9] Li X, Cheng K, Li X, Zhou Y, Liu J, Zeng H, et al. Phase I clinical trial of HC-1119: a deuterated form of enzalutamide. Int J Cancer. 2021;149:1473–82. 10.1002/ijc.33706 34109624

[10] Kaya-Okur HS, Wu SJ, Codomo CA, Pledger ES, Bryson TD, Henikoff JG, et al. CUT&Tag for efficient epigenomic profiling of small samples and single cells. Nat Commun. 2019;10:1930. 10.1038/s41467-019-09982-5 PMC6488672 31036827

[11] Bravo R, Frank R, Blundell PA, Macdonald-Bravo H. Cyclin/PCNA is the auxiliary protein of DNA polymerase-delta. Nature. 1987;326:515–7. 10.1038/326515a0 2882423

[12] Prelich G, Tan CK, Kostura M, Mathews MB, So AG, Downey KM, et al. Functional identity of proliferating cell nuclear antigen and a DNA polymerase-delta auxiliary protein. Nature. 1987;326:517–20. 10.1038/326517a0 2882424

[13] Lin YC, Liu D, Chakraborty A, Kadyrova LY, Song YJ, Hao Q, et al. Orc6 is a component of the replication fork and enables efficient mismatch repair. Proc Natl Acad Sci USA. 2022;119:e2121406119. 10.1073/pnas.2121406119 PMC9295755 35622890

[14] Cirillo LA, Lin FR, Cuesta I, Friedman D, Jarnik M, Zaret KS. Opening of compacted chromatin by early developmental transcription factors HNF3 (FoxA) and GATA-4. Mol Cell. 2002;9:279–89. 10.1016/s1097-2765(02)00459-8 11864602

[15] Gao N, Zhang J, Rao MA, Case TC, Mirosevich J, Wang Y, et al. The role of hepatocyte nuclear factor-3 alpha (Forkhead Box A1) and androgen receptor in transcriptional regulation of prostatic genes. Mol Endocrinol. 2003;17:1484–507. 10.1210/me.2003-0020 12750453

[16] Wang Q, Li W, Zhang Y, Yuan X, Xu K, Yu J, et al. Androgen receptor regulates a distinct transcription program in androgen-independent prostate cancer. Cell. 2009;138:245–56. 10.1016/j.cell.2009.04.056 PMC2726827 19632176

[17] Carroll JS, Liu XS, Brodsky AS, Li W, Meyer CA, Szary AJ, et al. Chromosome-wide mapping of estrogen receptor binding reveals long-range regulation requiring the forkhead protein FoxA1. Cell. 2005;122:33–43. 10.1016/j.cell.2005.05.008 16009131

[18] Hurtado A, Holmes KA, Ross-Innes CS, Schmidt D, Carroll JS. FOXA1 is a key determinant of estrogen receptor function and endocrine response. Nat Genet. 2011;43:27–33. 10.1038/ng.730 PMC3024537 21151129

[19] Takaku M, Grimm SA, Shimbo T, Perera L, Menafra R, Stunnenberg HG, et al. GATA3-dependent cellular reprogramming requires activation-domain dependent recruitment of a chromatin remodeler. Genome Biol. 2016;17:36. 10.1186/s13059-016-0897-0 PMC4769547 26922637

[20] Theodorou V, Stark R, Menon S, Carroll JS. GATA3 acts upstream of FOXA1 in mediating ESR1 binding by shaping enhancer accessibility. Genome Res. 2013;23:12–22. 10.1101/gr.139469.112 PMC3530671 23172872

[21] Robinson JL, Macarthur S, Ross-Innes CS, Tilley WD, Neal DE, Mills IG, et al. Androgen receptor driven transcription in molecular apocrine breast cancer is mediated by FoxA1. EMBO J. 2011;30:3019–27. 10.1038/emboj.2011.216 PMC3160190 21701558

[22] Hosseinzadeh L, Kikhtyak Z, Laven-Law G, Pederson SM, Puiu CG, D’Santos CS, et al. The androgen receptor interacts with GATA3 to transcriptionally regulate a luminal epithelial cell phenotype in breast cancer. Genome Biol. 2024;25:44. 10.1186/s13059-023-03161-y PMC10840202 38317241

[23] Mohammed H, Taylor C, Brown GD, Papachristou EK, Carroll JS, D’Santos CS. Rapid immunoprecipitation mass spectrometry of endogenous proteins (RIME) for analysis of chromatin complexes. Nat Protoc. 2016;11:316–26. 10.1038/nprot.2016.020 26797456

[24] Shao W, Keeton EK, McDonnell DP, Brown M. Coactivator AIB1 links estrogen receptor transcriptional activity and stability. Proc Natl Acad Sci USA. 2004;101:11599–604. 10.1073/pnas.0402997101 PMC511007 15289619

[25] Wang Q, Li W, Liu XS, Carroll JS, Janne OA, Keeton EK, et al. A hierarchical network of transcription factors governs androgen receptor-dependent prostate cancer growth. Mol Cell. 2007;27:380–92. 10.1016/j.molcel.2007.05.041 PMC3947890 17679089

[26] Lehmann BD, Bauer JA, Schafer JM, Pendleton CS, Tang L, Johnson KC, et al. PIK3CA mutations in androgen receptor-positive triple negative breast cancer confer sensitivity to the combination of PI3K and androgen receptor inhibitors. Breast Cancer Res. 2014;16:406. 10.1186/s13058-014-0406-x PMC4187324 25103565

[27] Fritsch C, Huang A, Chatenay-Rivauday C, Schnell C, Reddy A, Liu M, et al. Characterization of the novel and specific PI3Kalpha inhibitor NVP-BYL719 and development of the patient stratification strategy for clinical trials. Mol Cancer Ther. 2014;13:1117–29. 10.1158/1535-7163.MCT-13-0865 24608574

[28] Andre F, Ciruelos E, Rubovszky G, Campone M, Loibl S, Rugo HS, et al. Alpelisib for PIK3CA-mutated, hormone receptor-positive advanced breast cancer. N Engl J Med. 2019;380:1929–40. 10.1056/NEJMoa1813904 31091374

[29] Rampurwala M, Wisinski KB, O’Regan R. Role of the androgen receptor in triple-negative breast cancer. Clin Adv Hematol Oncol. 2016;14:186–93. PMC5221599 27058032

[30] Shastry M, Jacob S, Rugo HS, Hamilton E. Antibody-drug conjugates targeting TROP-2: Clinical development in metastatic breast cancer. Breast. 2022;66:169–77. 10.1016/j.breast.2022.10.007 PMC9614644 36302269

[31] Eri LM, Tveter KJ. A prospective, placebo-controlled study of the antiandrogen casodex as treatment for patients with benign prostatic hyperplasia. J Urol. 1993;150:90–4. 10.1016/s0022-5347(17)35406-x 7685428

[32] Scher HI, Fizazi K, Saad F, Taplin M-E, Sternberg CN, Miller K, et al. Increased survival with enzalutamide in prostate cancer after chemotherapy. N Engl J Med. 2012;367:1187–97. 10.1056/NEJMoa1207506 22894553

[33] Smith MR, Saad F, Chowdhury S, Oudard S, Hadaschik BA, Graff JN, et al. Apalutamide treatment and metastasis-free survival in prostate cancer. N Engl J Med. 2018;378:1408–18. 10.1056/NEJMoa1715546 29420164

[34] Fizazi K, Shore N, Tammela TL, Ulys A, Vjaters E, Polyakov S, et al. Darolutamide in nonmetastatic, castration-resistant prostate cancer. N Engl J Med. 2019;380:1235–46. 10.1056/NEJMoa1815671 30763142

[35] Dai C, Ellisen LW. Revisiting androgen receptor signaling in breast cancer. Oncologist. 2023;28:383–91. 10.1093/oncolo/oyad049 PMC10166165 36972361

[36] Zhao L, Han X, Lu J, McEachern D, Wang S. A highly potent PROTAC androgen receptor (AR) degrader ARD-61 effectively inhibits AR-positive breast cancer cell growth in vitro and tumor growth in vivo. Neoplasia. 2020;22:522–32. 10.1016/j.neo.2020.07.002 PMC7498667 32928363

[37] Cimino-Mathews A, Subhawong AP, Illei PB, Sharma R, Halushka MK, Vang R, et al. GATA3 expression in breast carcinoma: utility in triple-negative, sarcomatoid, and metastatic carcinomas. Hum Pathol. 2013;44:1341–9. 10.1016/j.humpath.2012.11.003 PMC3991123 23375642

[38] Zheng R, Blobel GA. GATA transcription factors and cancer. Genes Cancer. 2010;1:1178–88. 10.1177/1947601911404223 PMC3092280 21779441

[39] Kim S, Moon BI, Lim W, Park S, Cho MS, Sung SH. Expression patterns of GATA3 and the androgen receptor are strongly correlated in patients with triple-negative breast cancer. Hum Pathol. 2016;55:190–5. 10.1016/j.humpath.2016.04.013 27184484

[40] Mangia A, Saponaro C, Vagheggini A, Opinto G, Centonze M, Vicenti C, et al. Should tumor infiltrating lymphocytes, androgen receptor, and FOXA1 expression predict the clinical outcome in triple negative breast cancer patients? Cancers. 2019;11:1393. 10.3390/cancers11091393 PMC6769726 31540486

[41] Antonarakis ES. AR signaling in human malignancies: prostate cancer and beyond. Cancers. 2018;10:22. 10.3390/cancers10010022 PMC5789372 29346310

[42] Kanda T, Jiang X, Yokosuka O. Androgen receptor signaling in hepatocellular carcinoma and pancreatic cancers. World J Gastroenterol. 2014;20:9229–36. 10.3748/wjg.v20.i28.9229 PMC4110552 25071315

[43] Langmead B, Salzberg SL. Fast gapped-read alignment with Bowtie 2. Nat Methods. 2012;9:357–9. 10.1038/nmeth.1923 PMC3322381 22388286

[44] Li B, Dewey CN. RSEM: accurate transcript quantification from RNA-Seq data with or without a reference genome. BMC Bioinform. 2011;12:323. 10.1186/1471-2105-12-323 PMC3163565 21816040

[45] Love MI, Huber W, Anders S. Moderated estimation of fold change and dispersion for RNA-seq data with DESeq2. Genome Biol. 2014;15:550. 10.1186/s13059-014-0550-8 PMC4302049 25516281

[46] Yu G, Wang LG, Han Y, He QY. clusterProfiler: an R package for comparing biological themes among gene clusters. OMICS. 2012;16:284–7. 10.1089/omi.2011.0118 PMC3339379 22455463

[47] Bareche Y, Venet D, Ignatiadis M, Aftimos P, Piccart M, Rothe F, et al. Unravelling triple-negative breast cancer molecular heterogeneity using an integrative multiomic analysis. Ann Oncol. 2018;29:895–902. 10.1093/annonc/mdy024 PMC5913636 29365031

[48] Li H, Handsaker B, Wysoker A, Fennell T, Ruan J, Homer N, et al. The sequence alignment/map format and SAMtools. Bioinformatics. 2009;25:2078–9. 10.1093/bioinformatics/btp352 PMC2723002 19505943

[49] Zhang Y, Liu T, Meyer CA, Eeckhoute J, Johnson DS, Bernstein BE, et al. Model-based analysis of ChIP-Seq (MACS). Genome Biol. 2008;9:R137. 10.1186/gb-2008-9-9-r137 PMC2592715 18798982

[50] Ramirez F, Dundar F, Diehl S, Gruning BA, Manke T. deepTools: a flexible platform for exploring deep-sequencing data. Nucleic Acids Res. 2014;42:W187–91. 10.1093/nar/gku365 PMC4086134 24799436

[51] Robinson JT, Thorvaldsdottir H, Winckler W, Guttman M, Lander ES, Getz G, et al. Integrative genomics viewer. Nat Biotechnol. 2011;29:24–6. 10.1038/nbt.1754 PMC3346182 21221095

[52] Heinz S, Benner C, Spann N, Bertolino E, Lin YC, Laslo P, et al. Simple combinations of lineage-determining transcription factors prime cis-regulatory elements required for macrophage and B cell identities. Mol Cell. 2010;38:576–89. 10.1016/j.molcel.2010.05.004 PMC2898526 20513432

