# Supplementary material for: Constitutive androgen receptor activity sustains DNA replication programs in triple-negative breast cancer
Source: Cell Death Dis. 2026 Aug 6;17(1):917. doi: 10.1038/s41419-026-09180-4 (PMC13719998; doi:10.1038/s41419-026-09180-4)

Related to Fig.1A

Fig. 1A AR

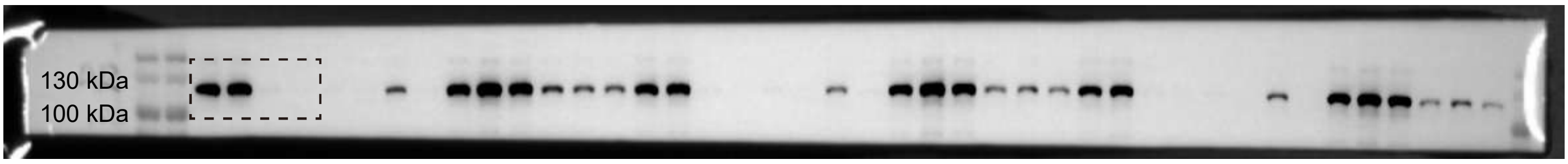

Fig. 1A GAPDH

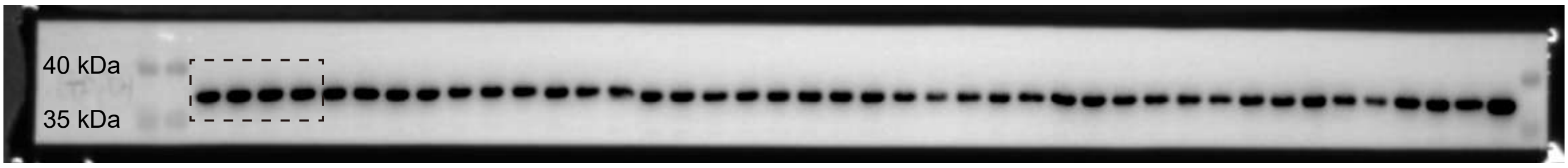

Related to Fig. 4B

Fig. 4B AR

130 kDa
100 kDa

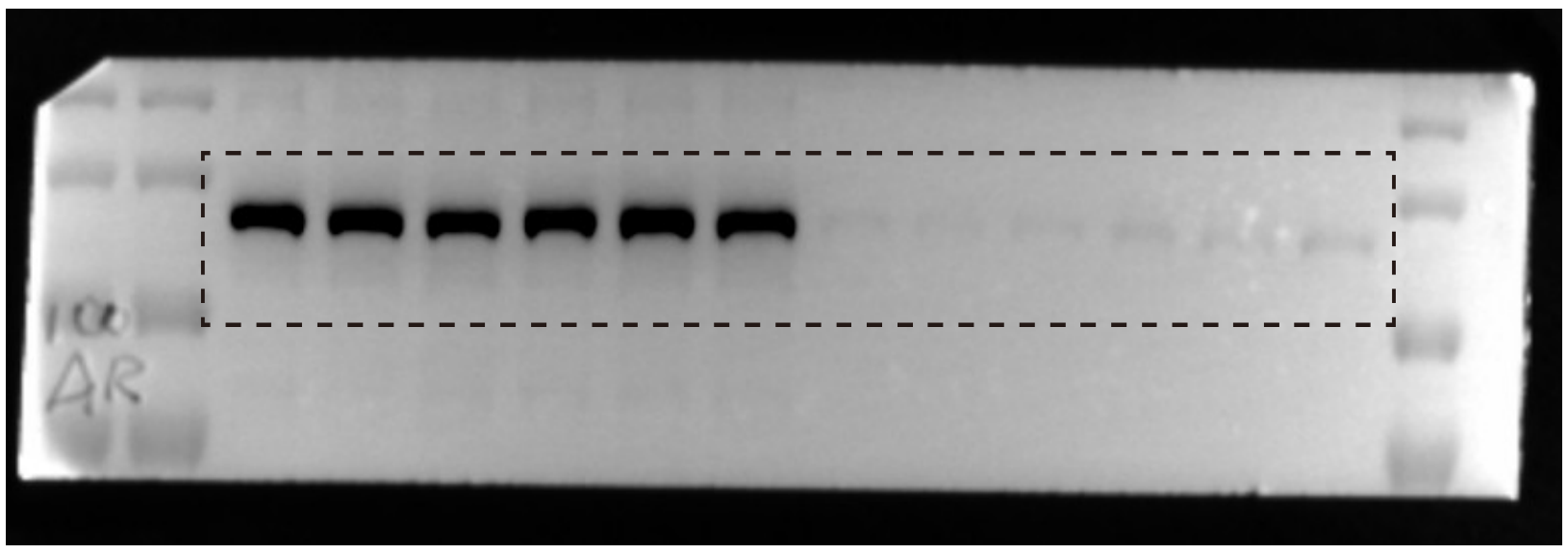

Fig. 4B PCNA

40 kDa
35 kDa

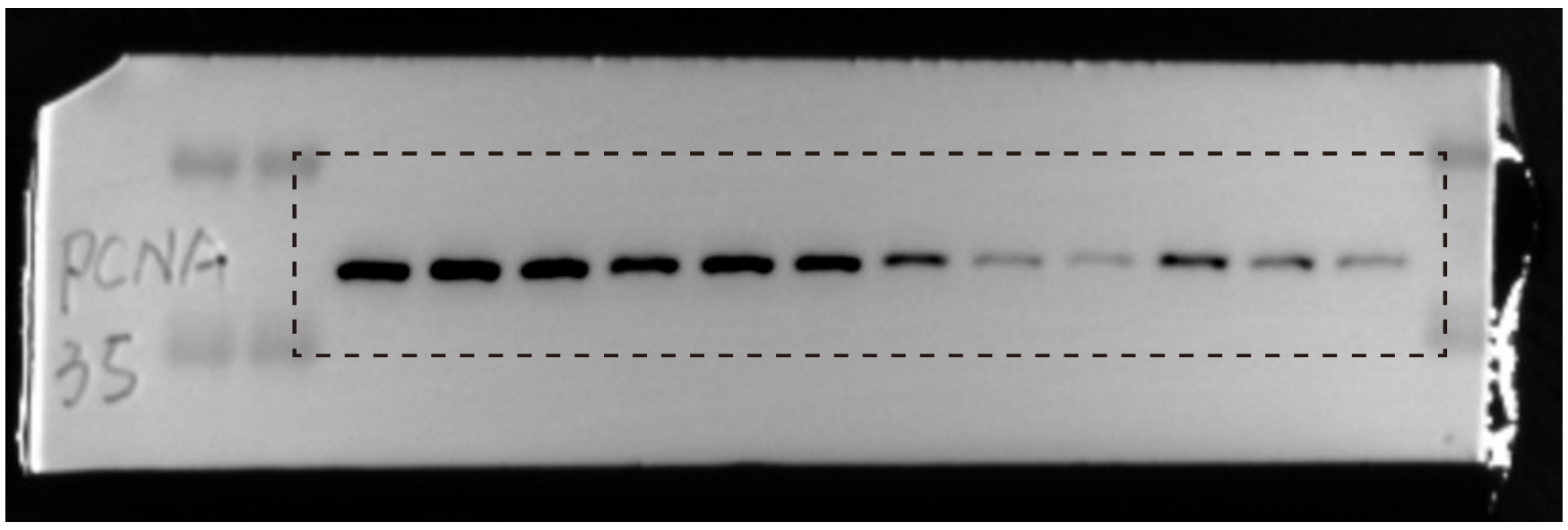

Fig. 4B ORC6

35 kDa
25 kDa

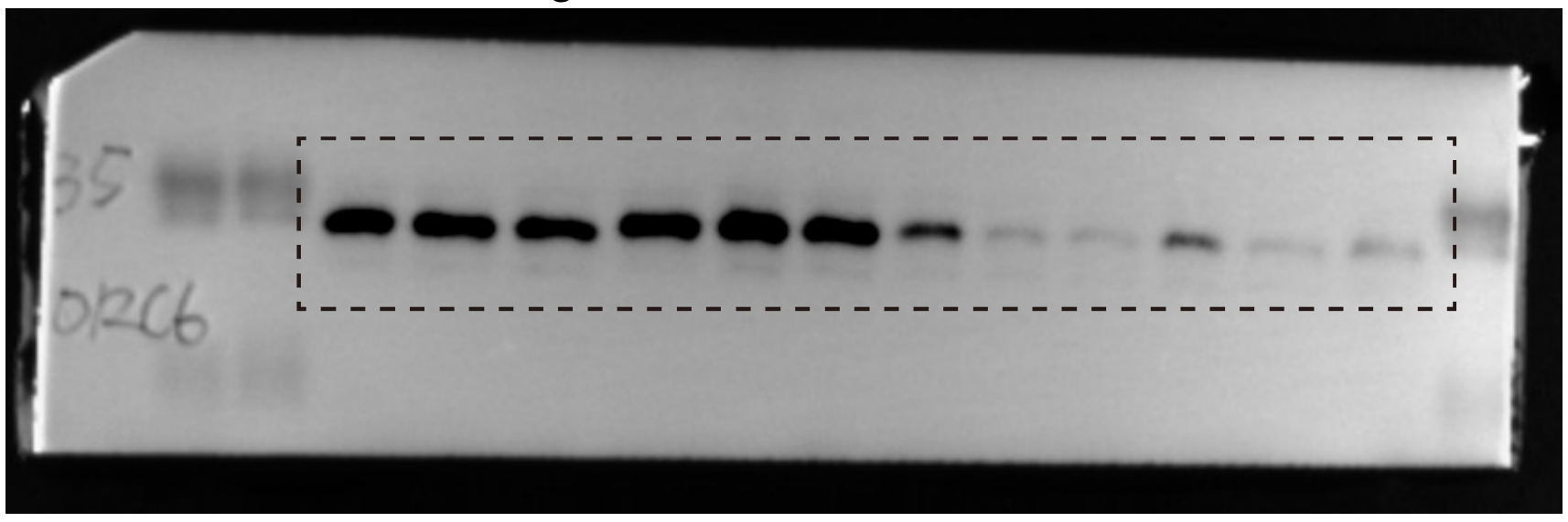

Fig. 4B GAPDH

40 kDa
35 kDa

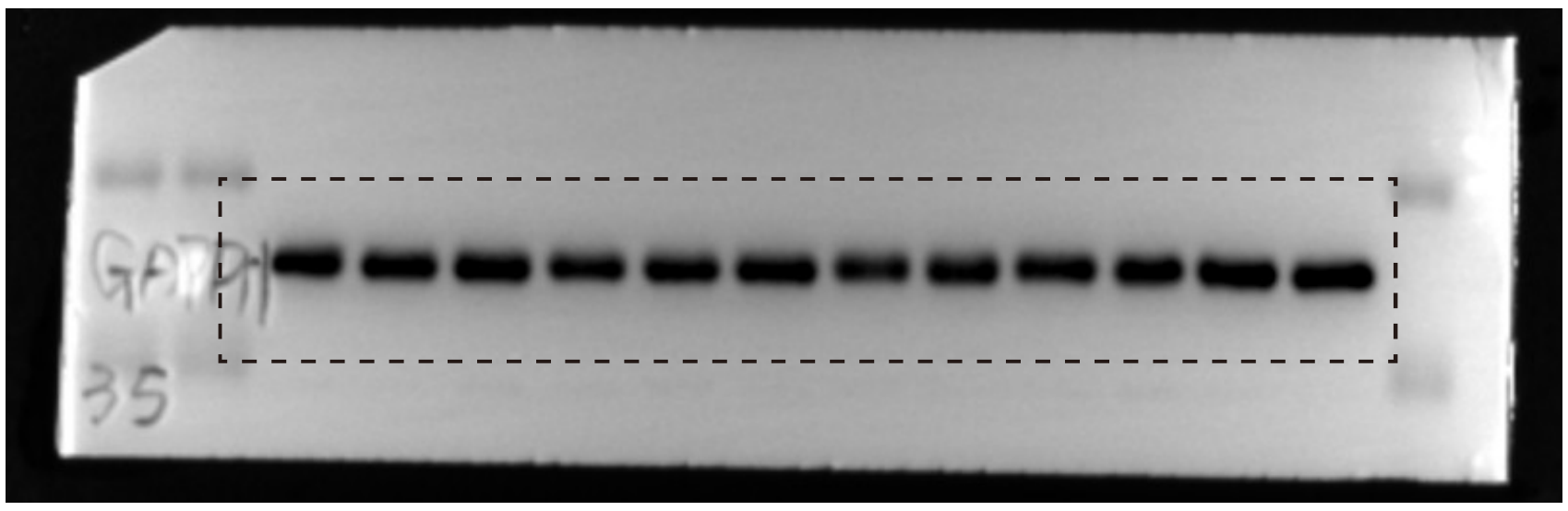

Related to Fig.4D

Fig. 4D AR

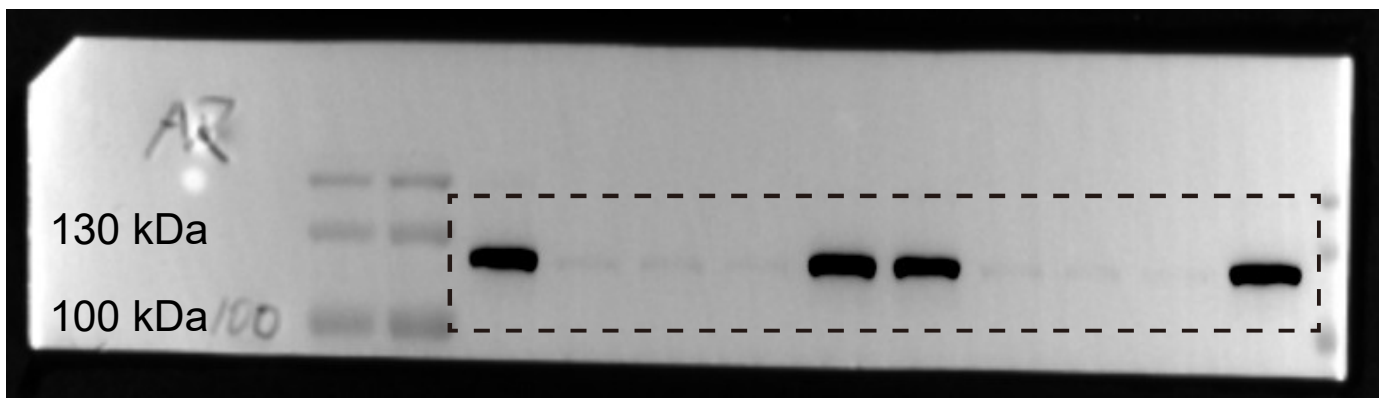

Fig. 4D PCNA

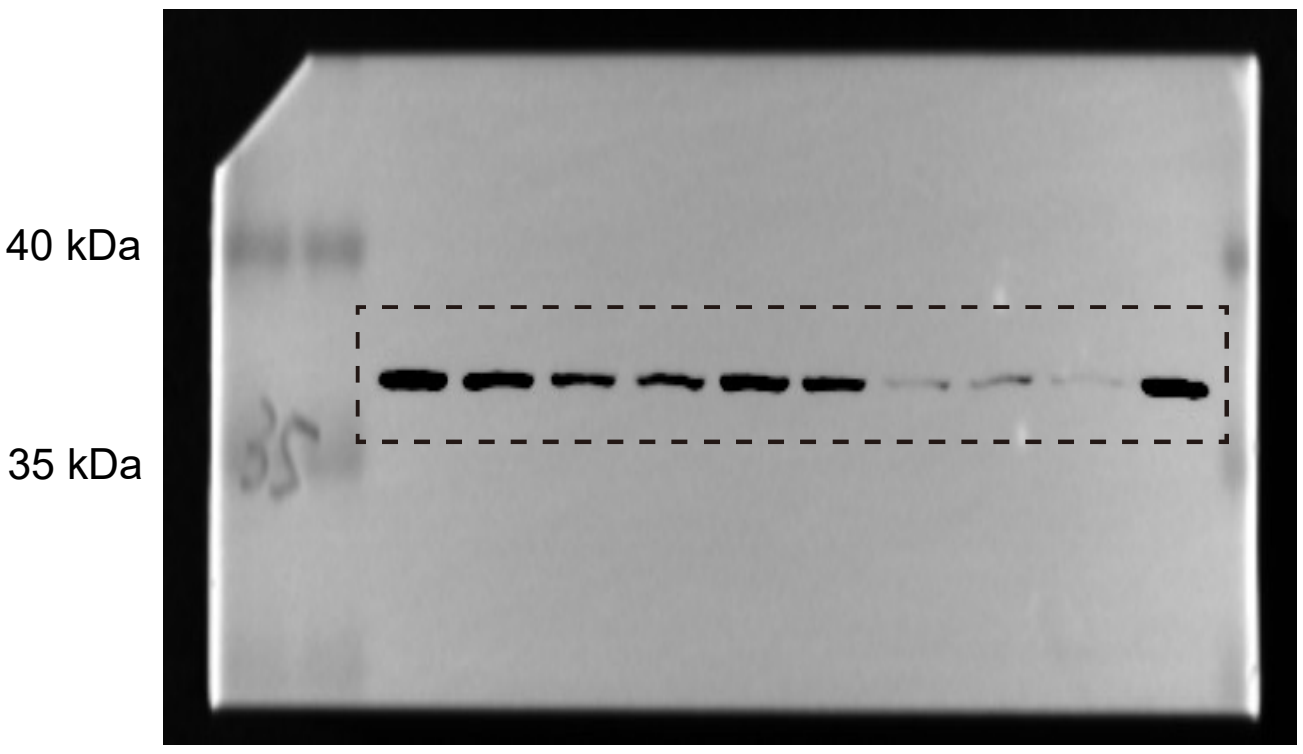

Fig. 4D ORC6

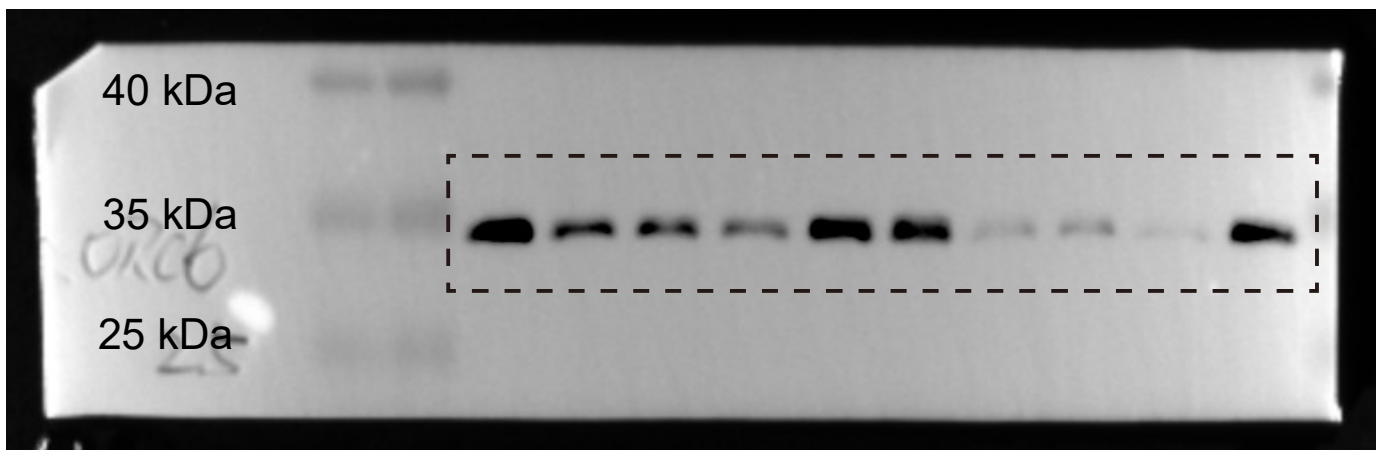

Fig. 4D GAPDH

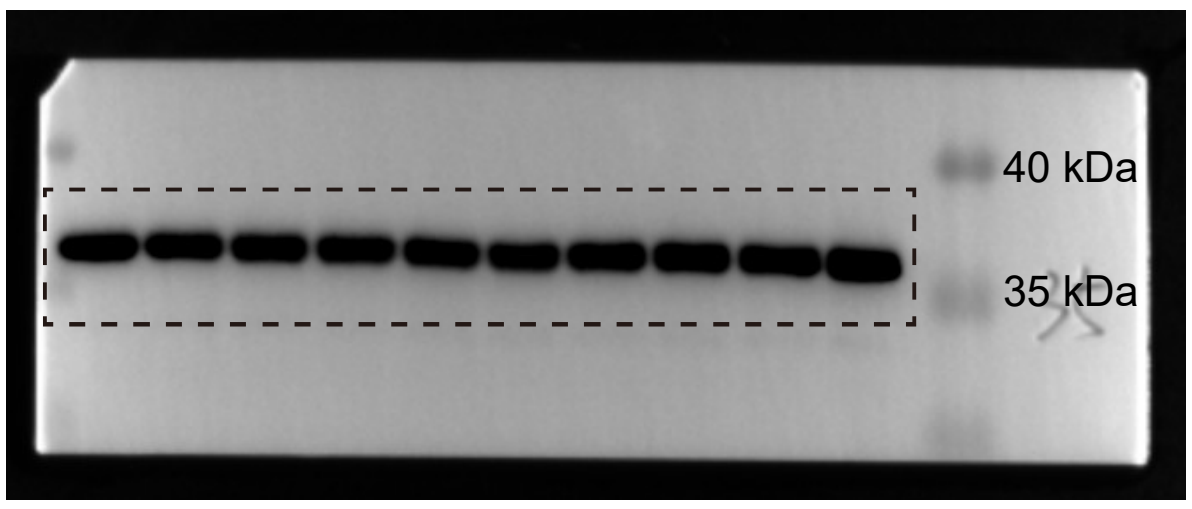

Related to Fig. 5B

Fig. 5B FoxA1

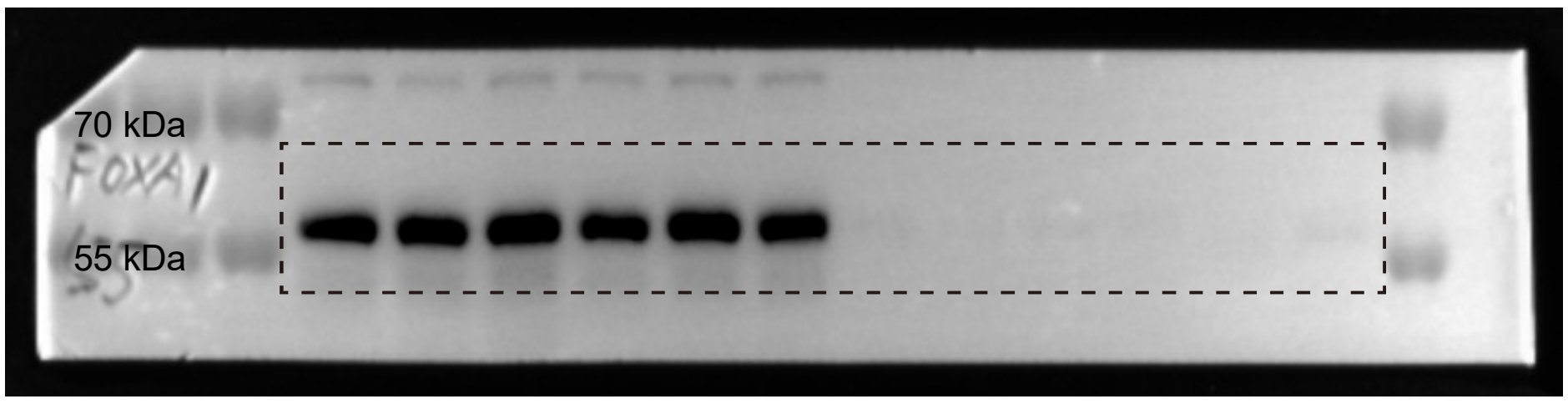

Fig. 5B PCNA

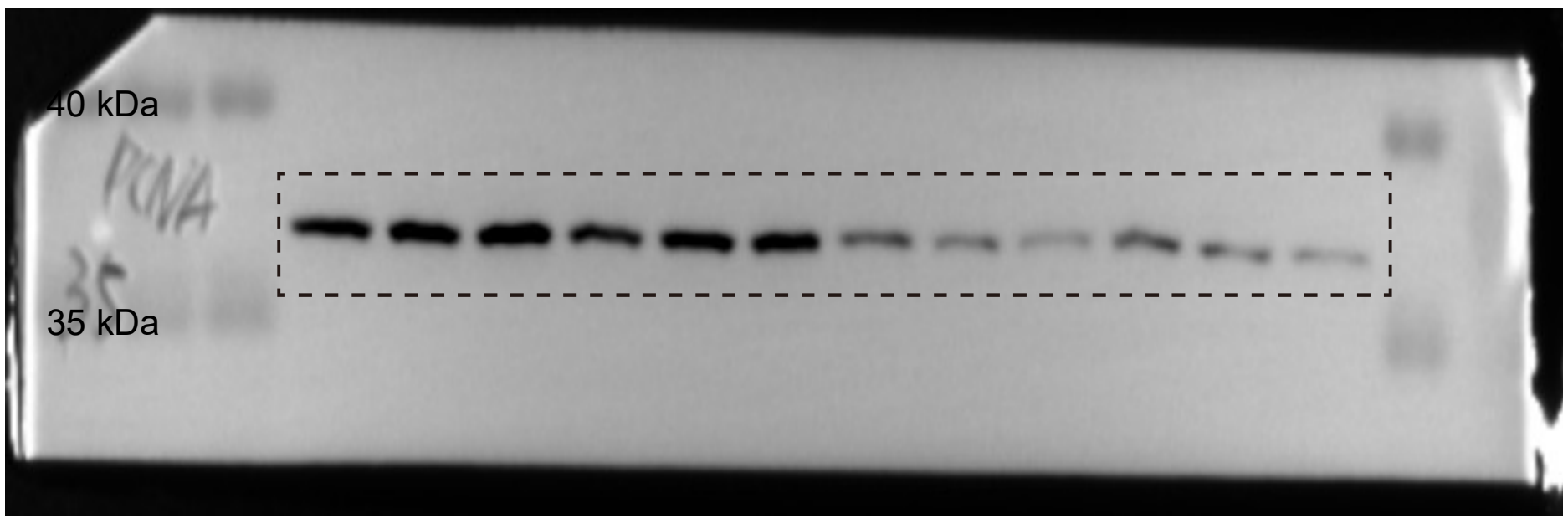

Fig. 5B ORC6

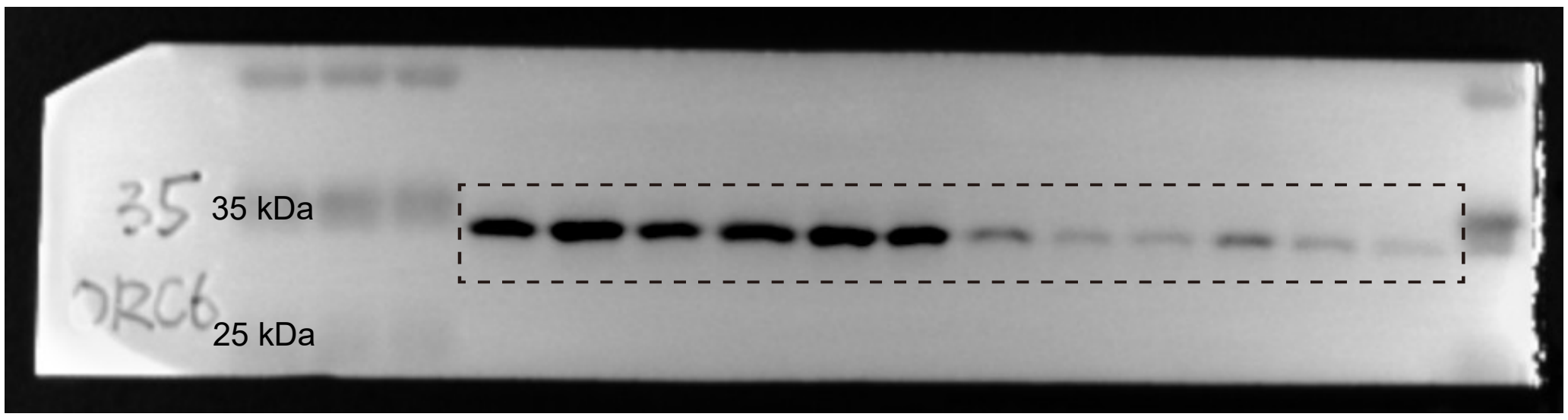

Fig. 5B GAPDH

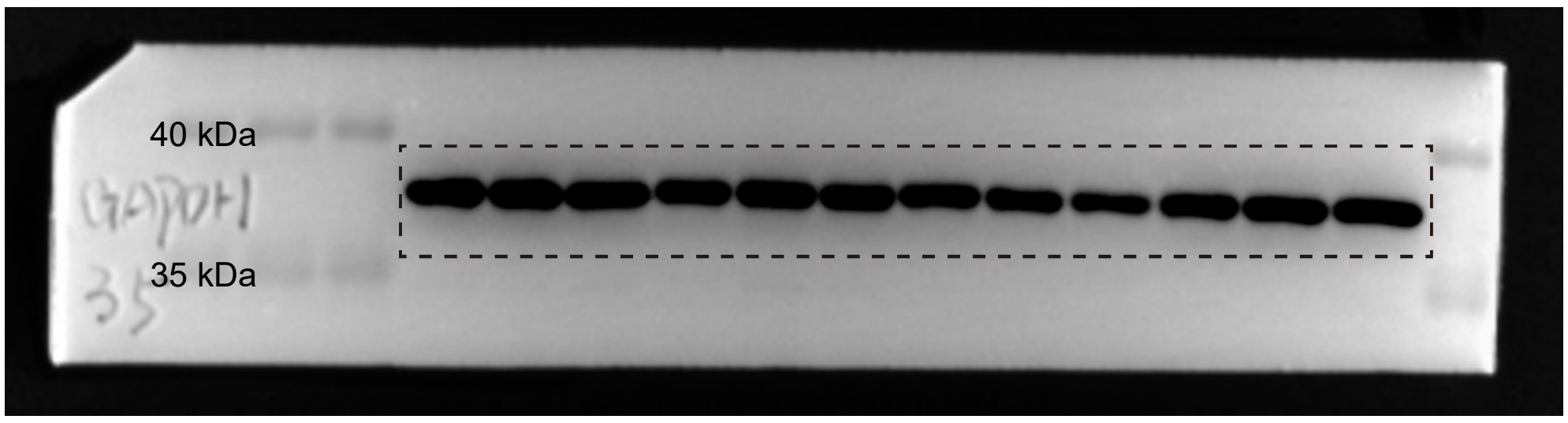

Related to Fig. 5C

Fig. 5C GATA3

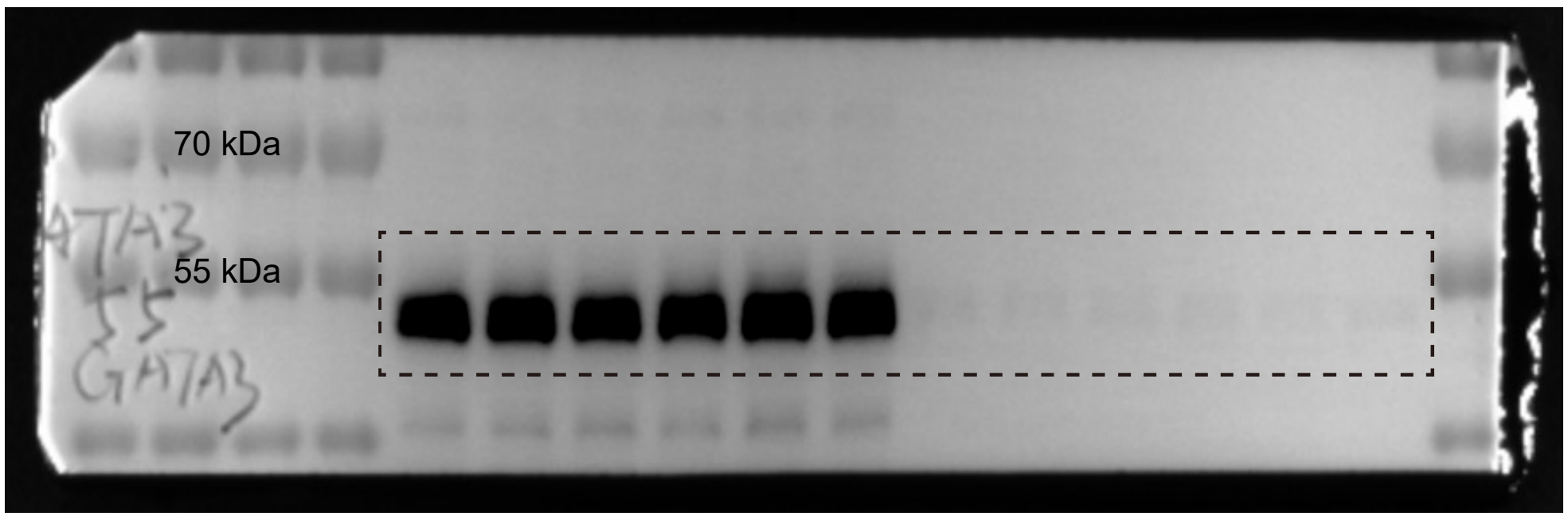

Fig. 5C PCNA

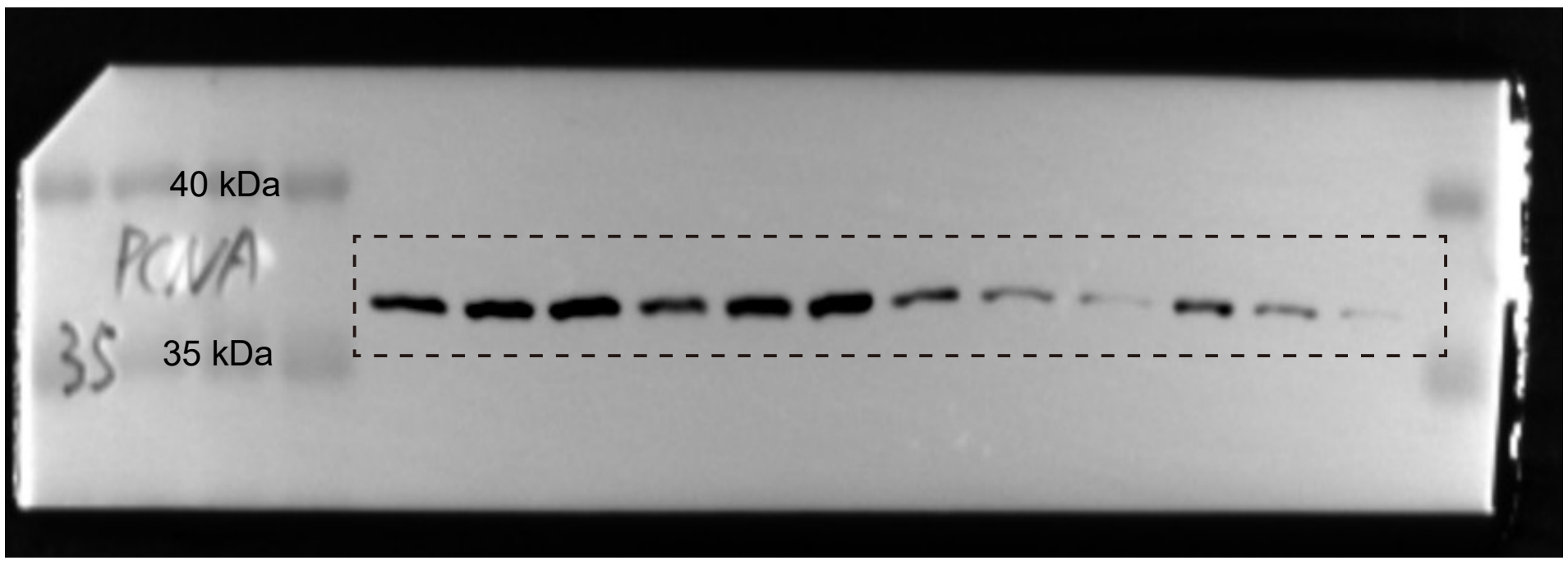

Fig. 5C ORC6

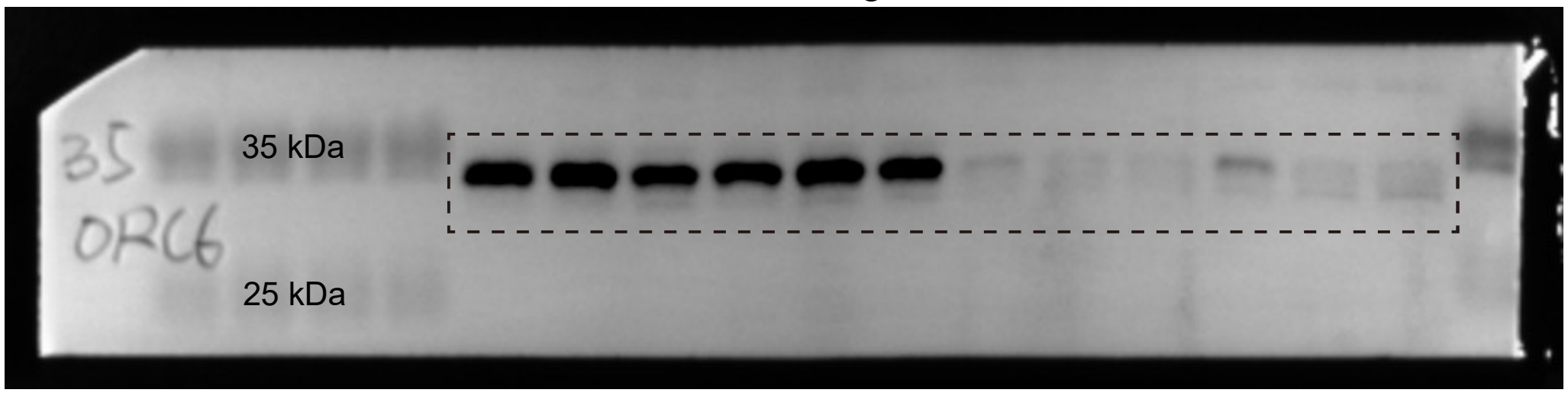

Fig. 5C GAPDH

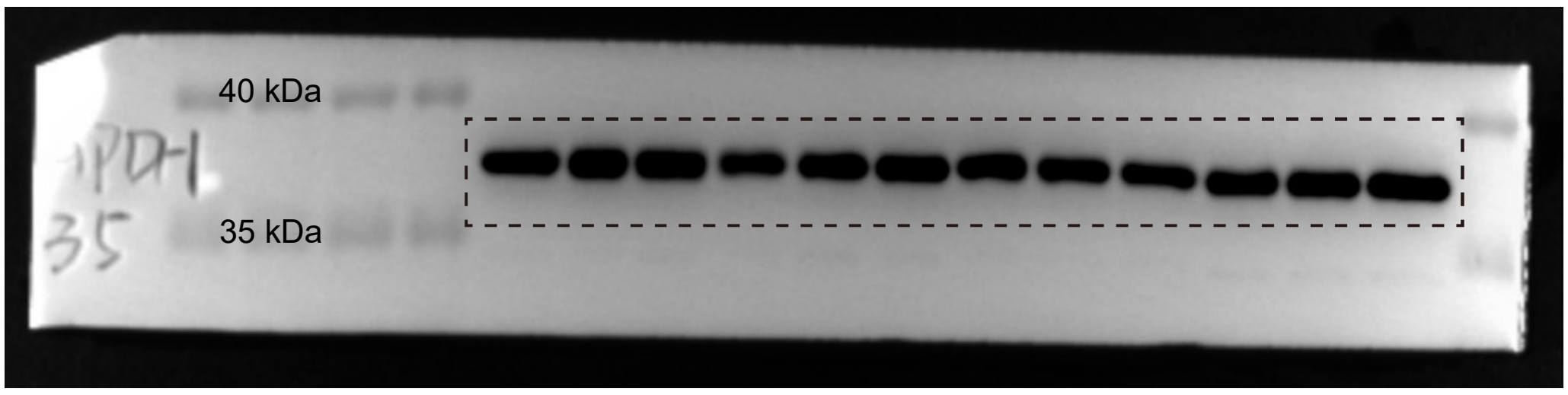

Related to Fig. 5E

Fig. 5E Biotin

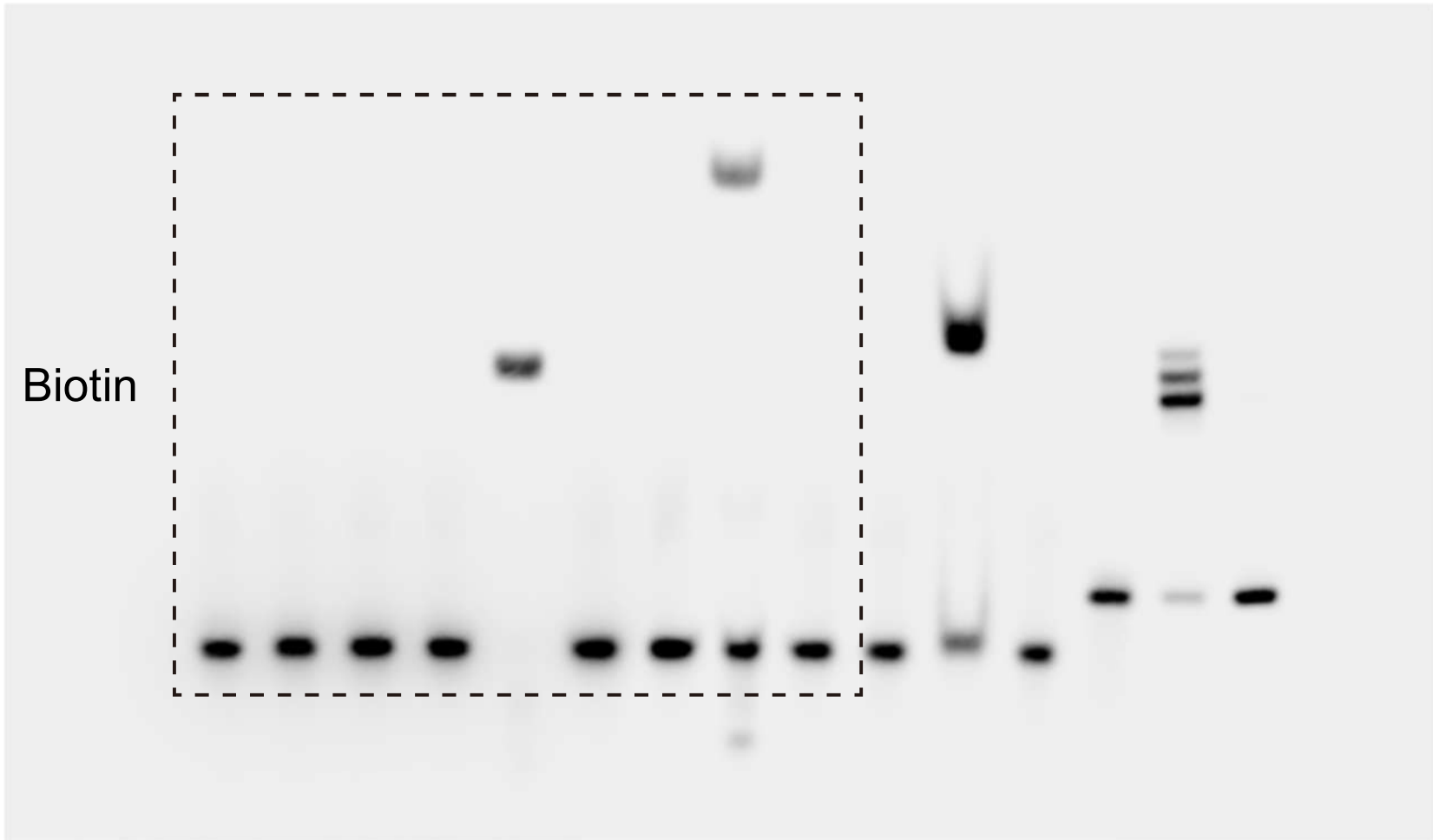

Related to Fig. S1

Fig. S1 AR

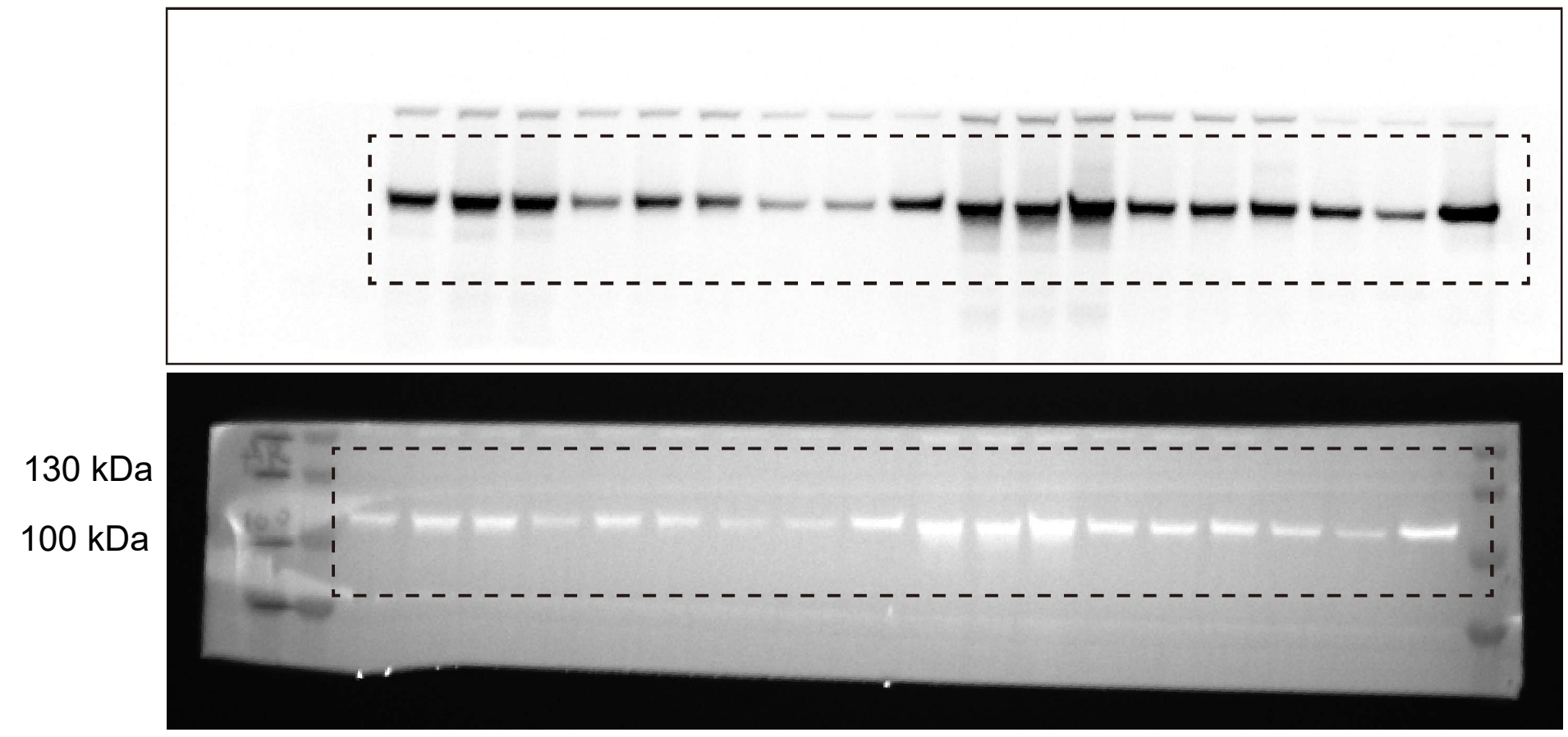

Fig. S1 Lamin B1

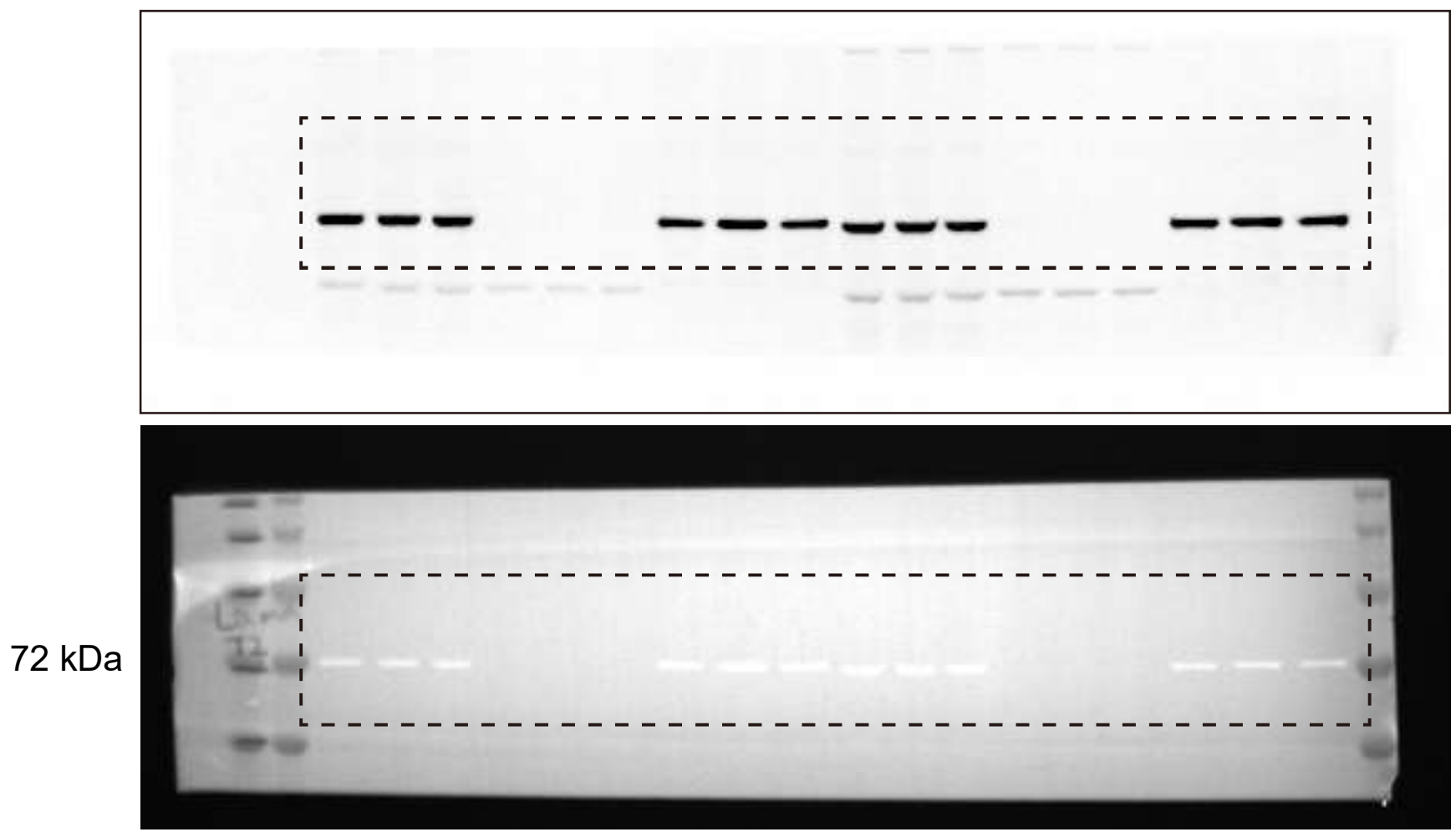

Fig. S1 GAPDH

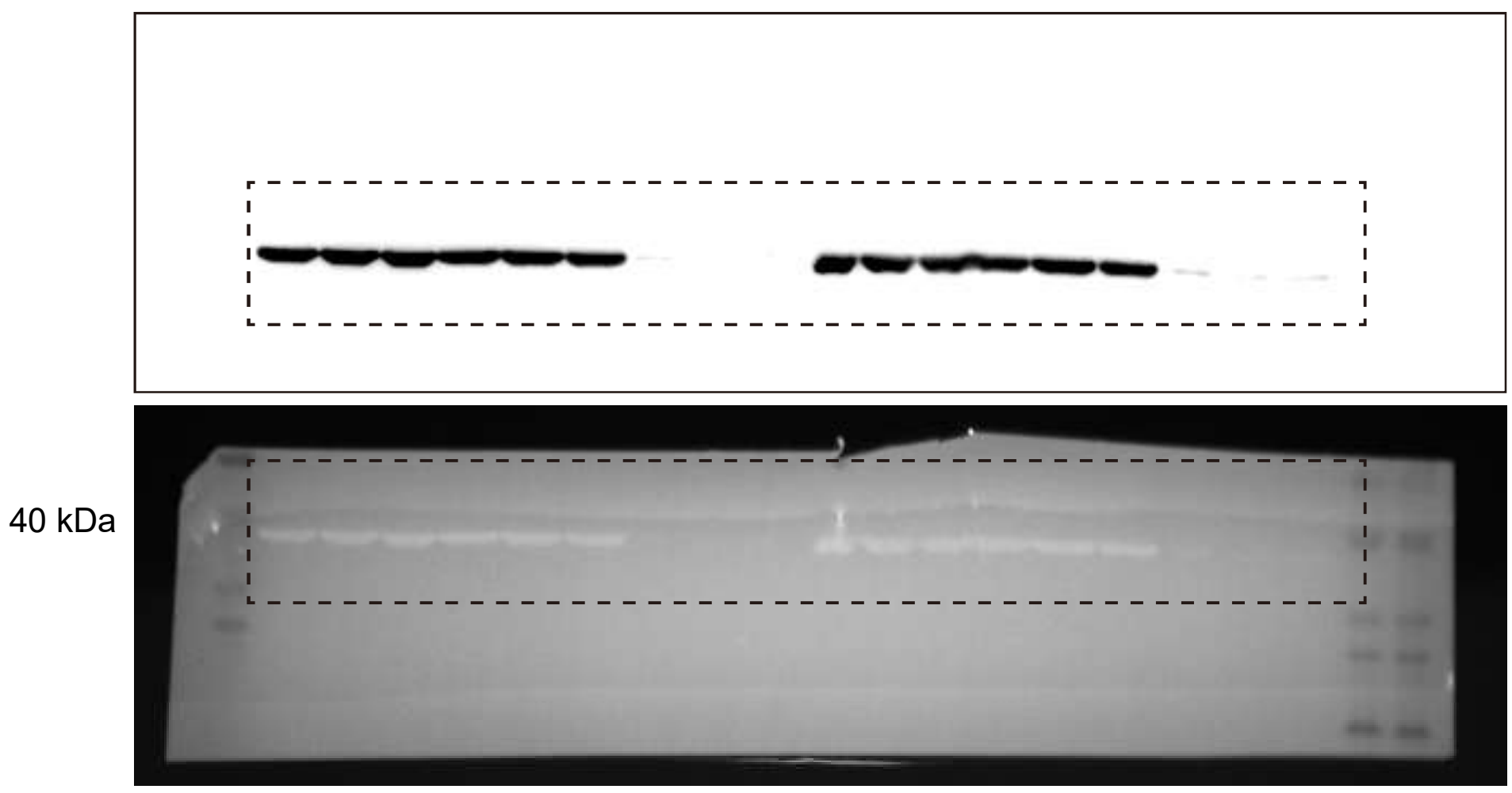

Related to Fig. S2

Fig. S2 AR

130 kDa
100 kDa

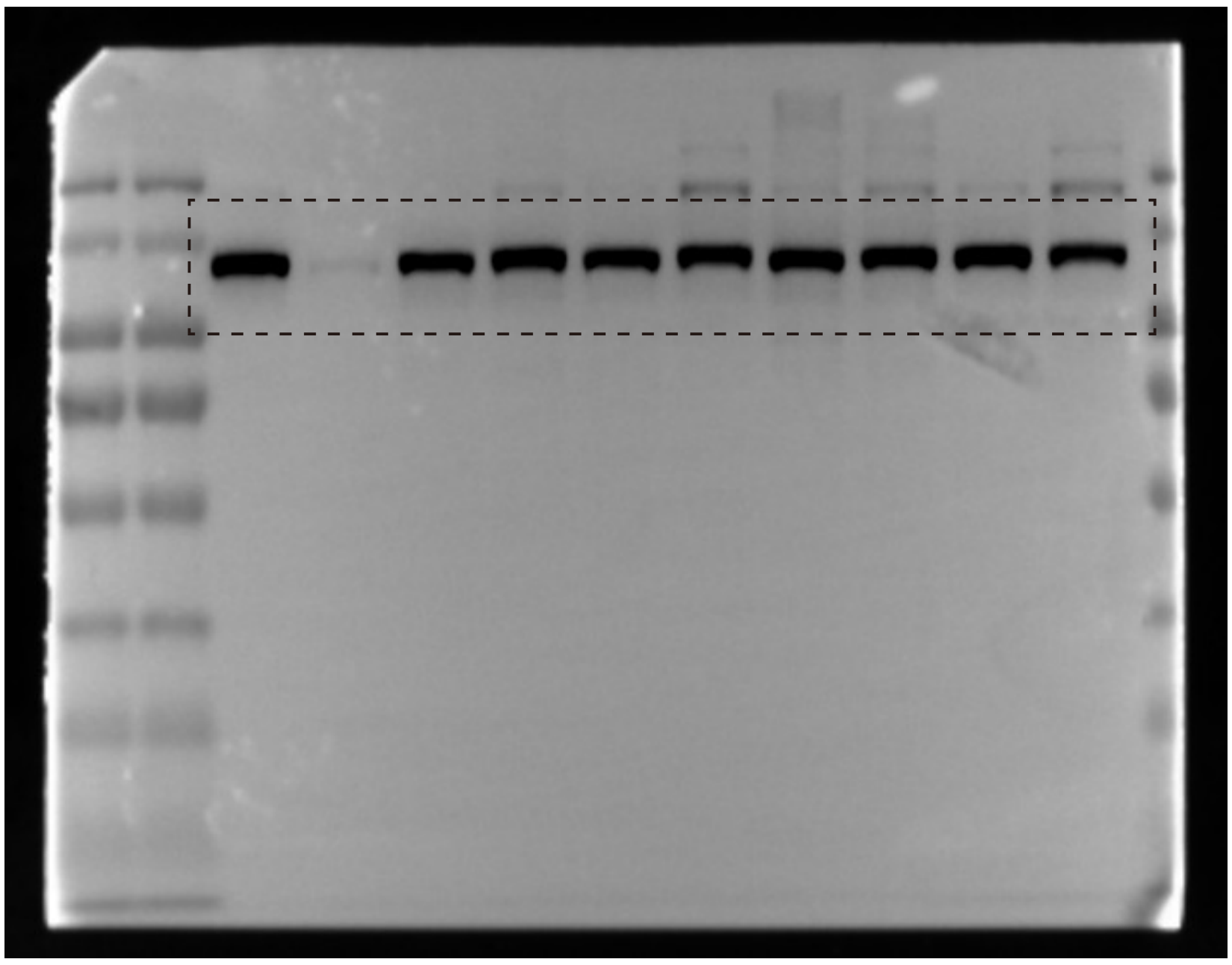

Fig. S2 GAPDH

40 kDa
35 kDa

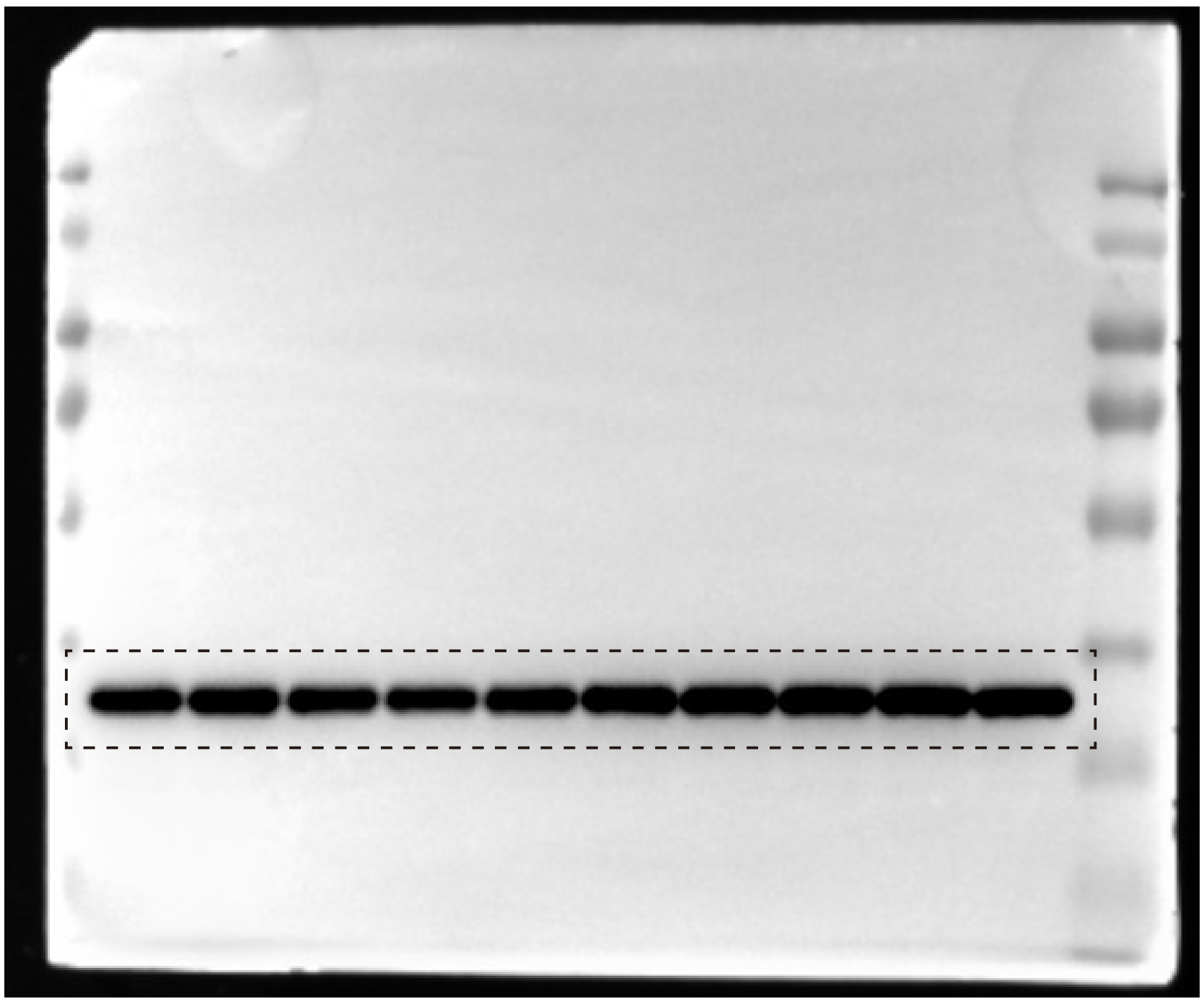

Related to Fig. S6A

Fig. S6A AR

130 kDa
100 kDa

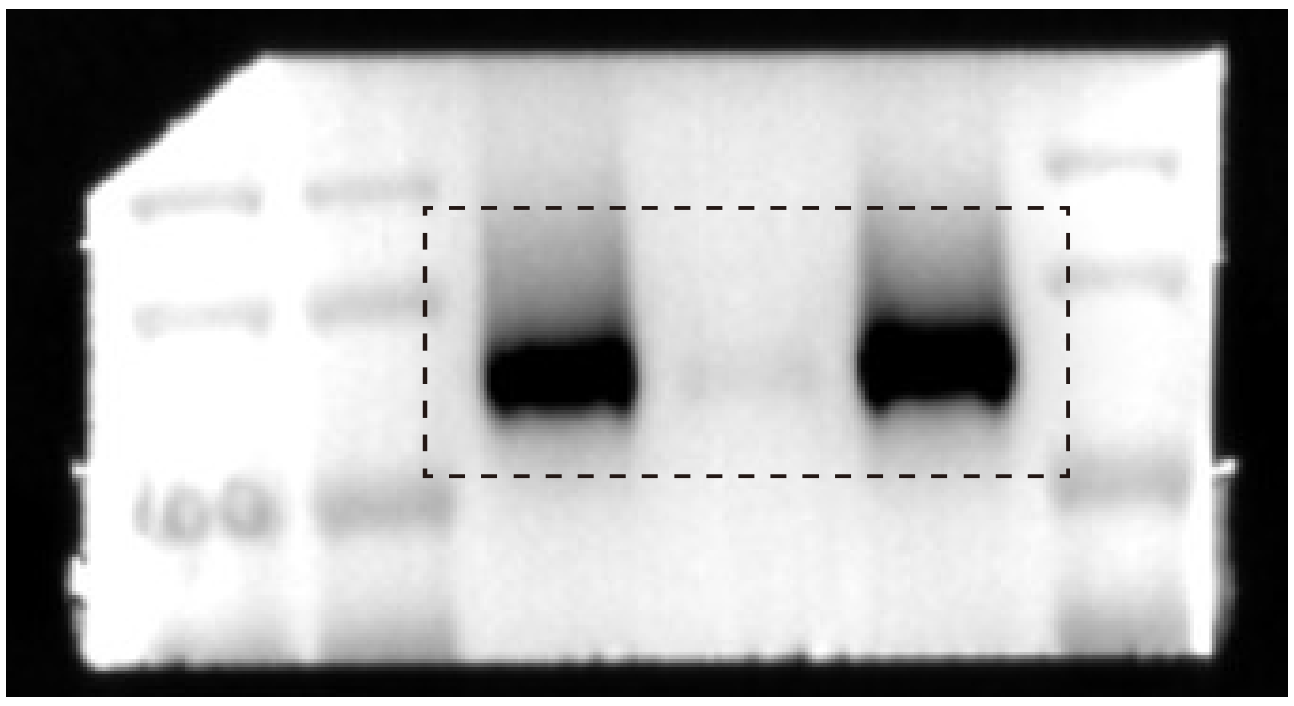

Fig. S6A GAPDH

40 kDa
35 kDa

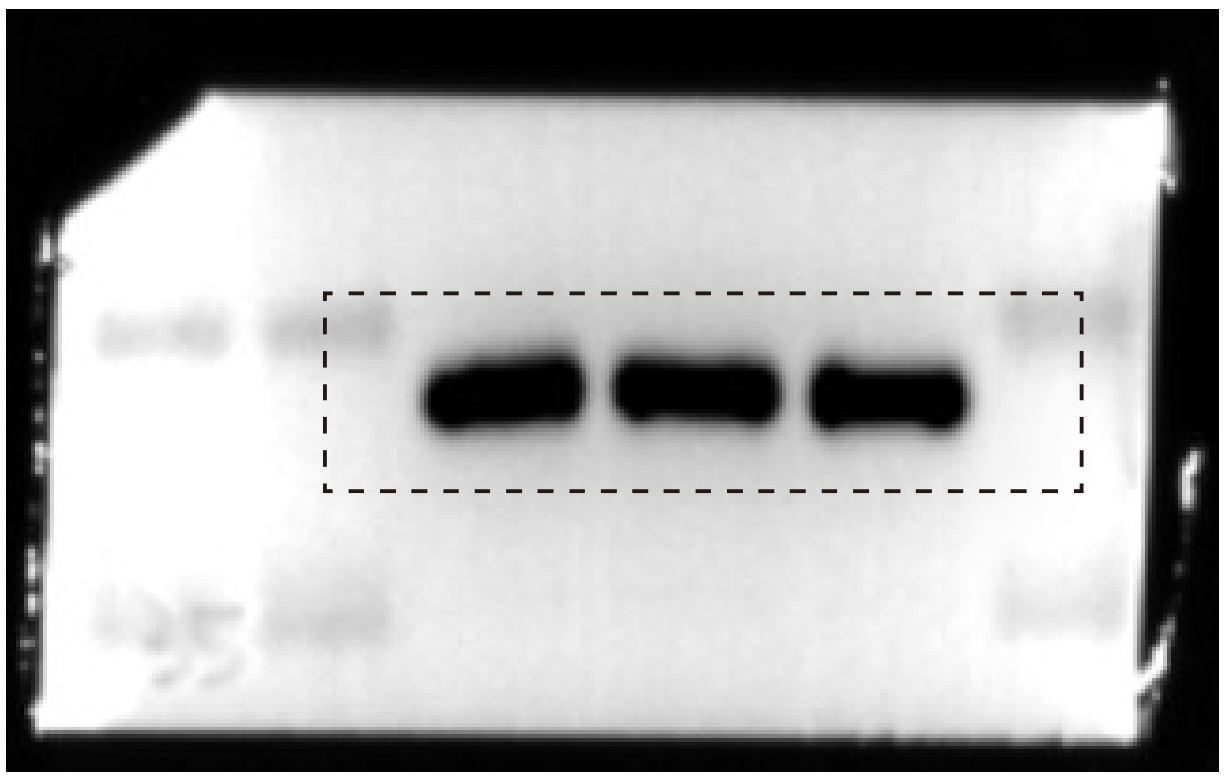

Related to Fig. S8B

Fig. S8B AR

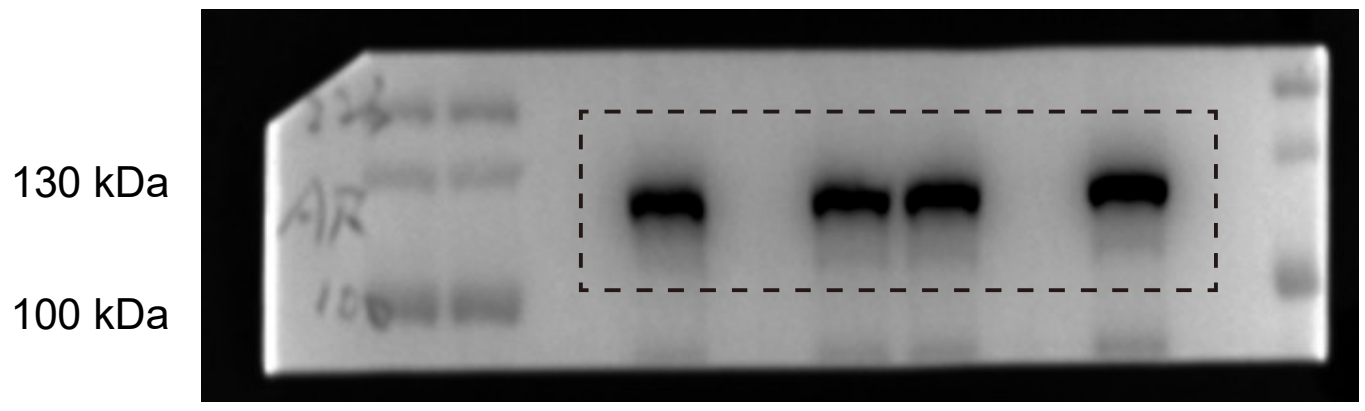

Fig. S8B PCNA

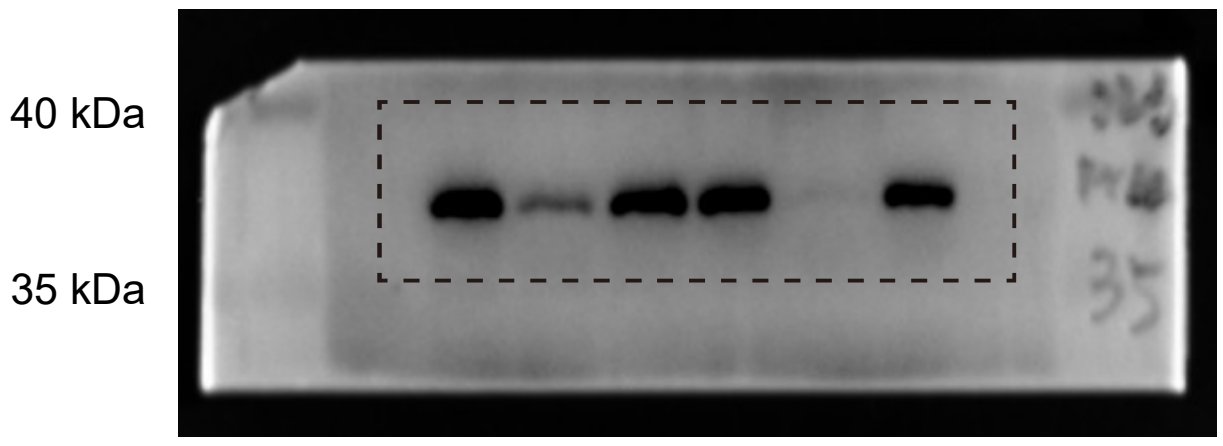

Fig. S8B ORC6

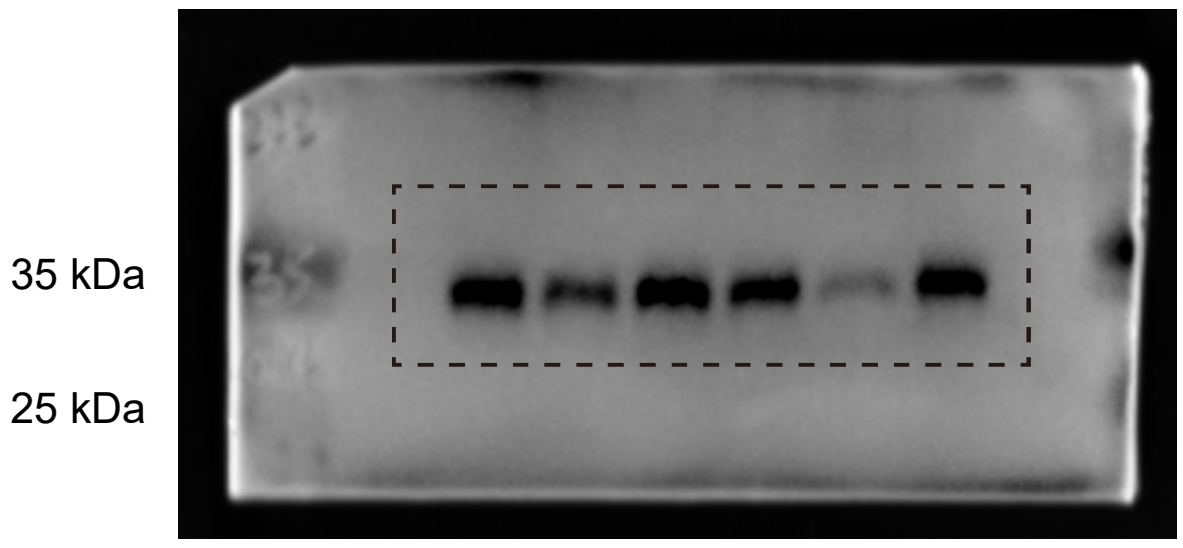

Fig. S8B GAPDH

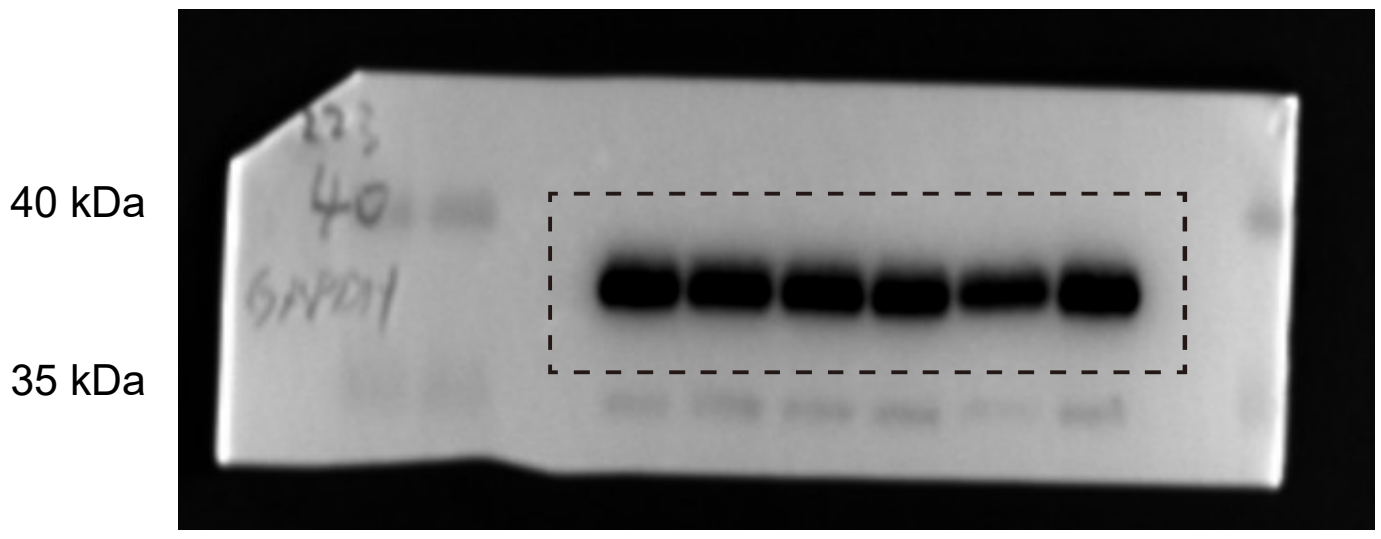

Related to Fig. S13

Fig. S13B

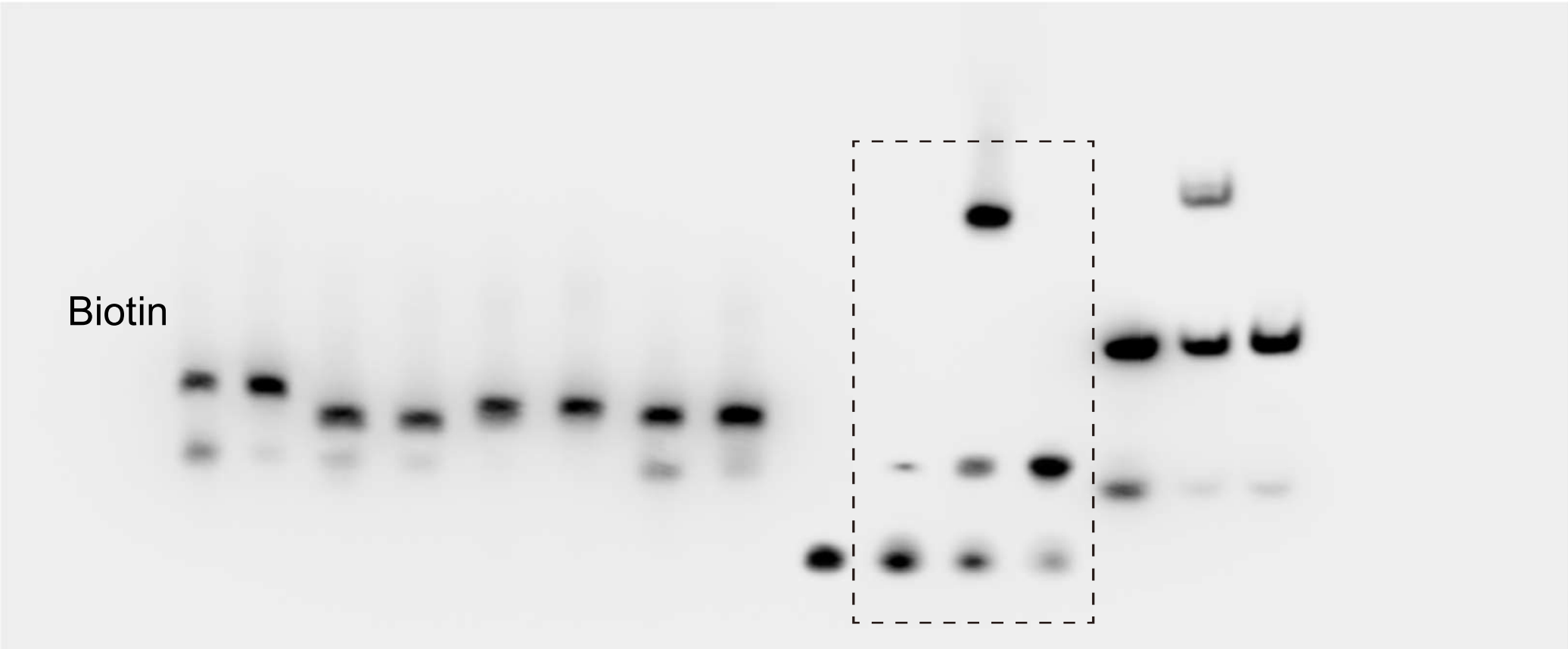

Fig. S13C

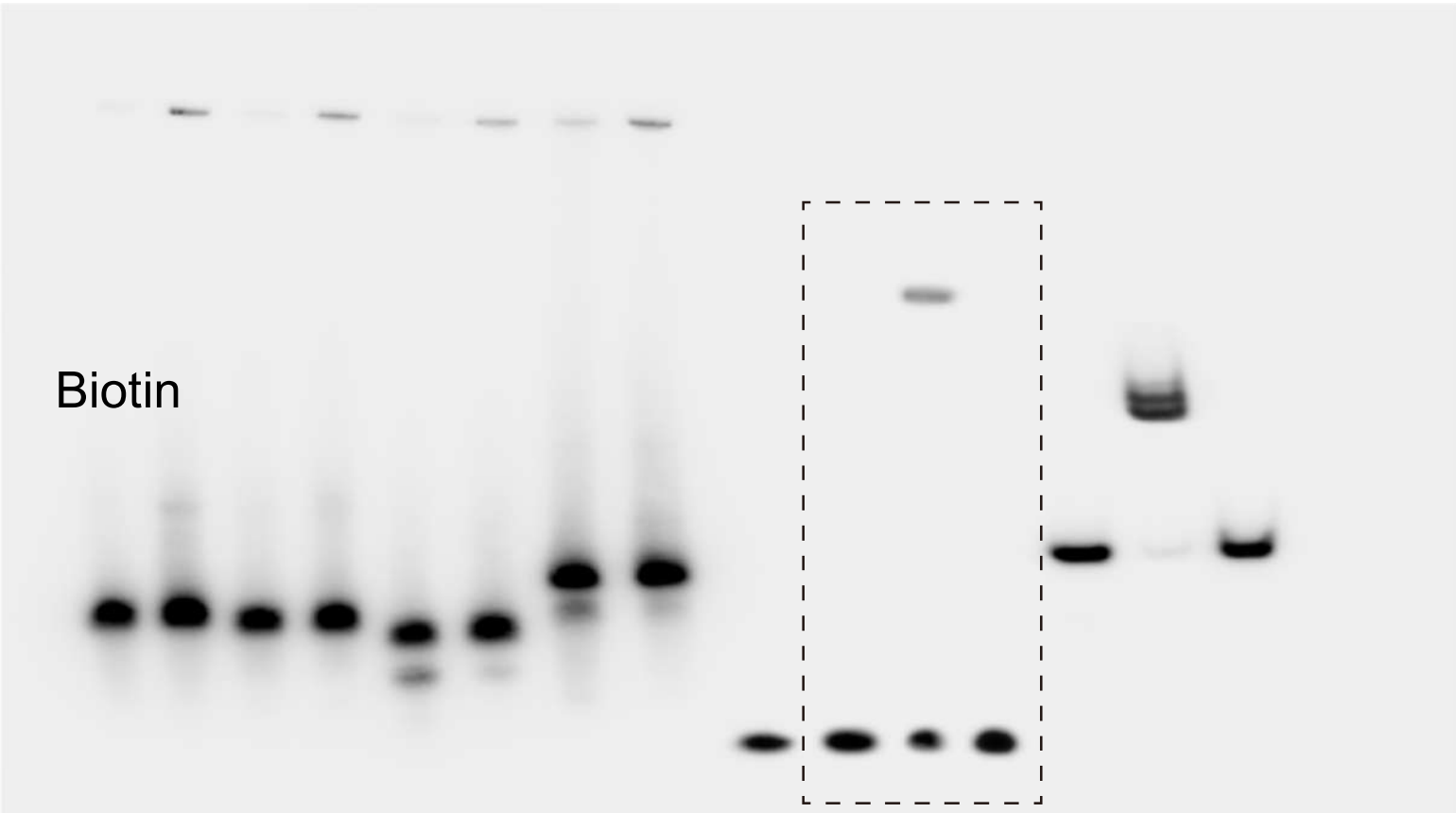

Fig. S13D

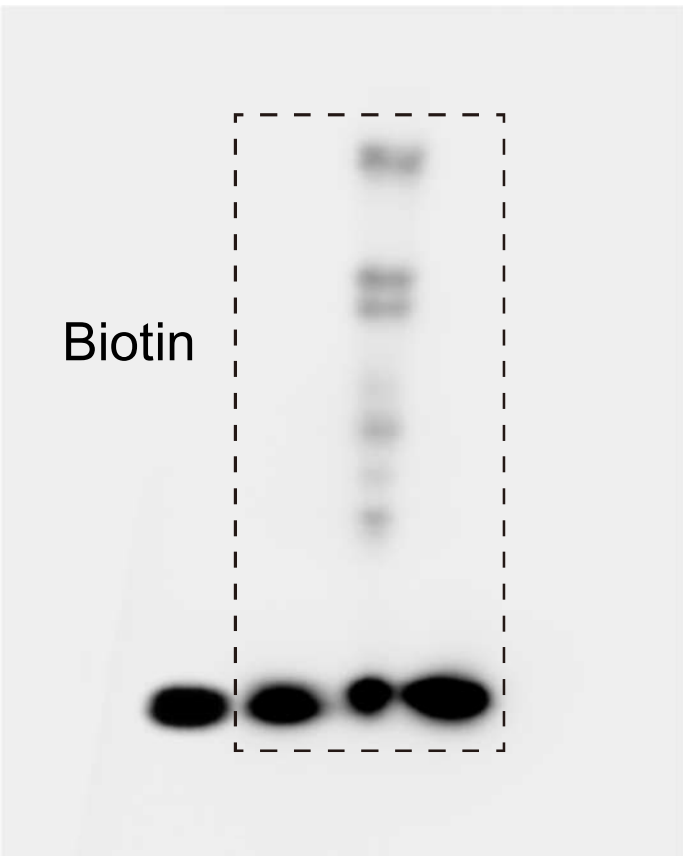

Supplement: Supplementary file 1 [file 41419_2026_9180_MOESM5_ESM.pdf]
