# Supplementary material for: Constitutive androgen receptor activity sustains DNA replication programs in triple-negative breast cancer
Source: Cell Death Dis. 2026 Aug 6;17(1):917. doi: 10.1038/s41419-026-09180-4 (PMC13719998; doi:10.1038/s41419-026-09180-4)
Supplement: Supplementary file 3 [file 41419_2026_9180_MOESM4_ESM.docx]

Supplementary Materials for

**Constitutive Androgen Receptor Activity Sustains DNA Replication Programs in Triple-Negative Breast Cancer**

**Authors**: Jing Li^†^, Hongwei Yuan^†^, Kefan Chen, Jian Peng, Wu Du, Xinghai Li^*^

†These authors contributed equally

**Affiliations**:

Hinova Pharmaceuticals Inc, Chengdu 610041, China

*Correspondence to: xhli@hinovapharma.com

**The file includes:**

Figs. S1 to S16

Tables S1 to S3 (provided separately)

**Fig. S1**

**Fig. S1. R1881 induces nuclear accumulation of AR in TNBC cells.** Immunoblots of AR expression in total cell lysates, cytoplasmic, and nuclear fractions of MFM-223 and MDA-MB-453 cells. Cells were cultured in medium supplemented with full fetal bovine serum (FBS) or charcoal-stripped serum (CSS) harboring DMSO or 100 nM of R1881 for 24 h. Lamin B1 and GAPDH were used as loading controls for the nuclear and cytoplasmic fractions, respectively. Data are representative of three independent experiments.

**Fig. S2**

**Fig. S2. HP518-induced AR degradation is dependent on the ubiquitin-proteasome system in TNBC cells.** Immunoblots of AR in MDA-MB-453 cells that were pre-treated with 1 μM MLN7243, 1 μM MLN4924, 10 μM MG132, or 1 μM LEN (lenalidomide) for 2 h and then treated with 100 nM HP518 for 6 h.

**Fig. S3**

**Fig. S3. *AR* knockdown suppresses the colony-forming ability in TNBC cells.** Representative images of the colony formation assay in MDA-MB-453 (upper panel) and MFM-223 (lower panel) cells. Cells were transfected with control siRNA (siCtrl) or two independent siRNAs targeting *AR* (*AR* si-1 and *AR* si-2). Mock-treated cells served as the negative control. After 14 days of incubation, colonies were fixed and visualized by crystal violet staining. Data are representative of three independent experiments.

**Fig. S4**

**Fig. S4. Anti-proliferative activity of HP518 is independent on androgens in AR-positive TNBC cells.** Drug sensitivity of MDA-MB-453 cells cultured in medium supplemented with charcoal-stripped FBS (CSS) in the presence or absence of R1881 after 5 days treatment with HP518. Data expressed as mean ± SD (n = 3).

**Fig. S5**

**Fig. S5. Representative AR immunohistochemical (IHC) images in the indicated TNBC patient samples.** Scale bar, 30 μm.

**Fig. S6**

**Fig. S6. The role of AR in TNBC is distinct from prostate cancer.** (**A**) Immunoblots of AR in MDA-MB-453 cells treated with vehicle, 100 nM HP518, or 1 µM HC-1119 for 24 h. (**B, C**) Relative mRNA expression of the well-known indicated androgen-dependent (B) and androgen-independent (C) AR-regulated genes in MDA-MB-453 and LNCaP cells treated with 100 nM HP518 or 1 µM HC-1119 versus vehicle for 24 h. Data expressed as mean ± SD (n = 3).

**Fig. S7**

**Fig. S7. Transcriptome analysis in MDA-MB-453 cells.** (**A, B**) Volcano plots depicting differentially expressed genes (computed by DESeq2) in MDA-MB-453 cells treated with 100 nM HP518 (A) or 1 µM HC-1119 (B) versus vehicle for 24 h. n = 2. Genes differentially expressed (log2(compound/vehicle) < -1 or > 1 and *P* value < 0.01) were labeled as red dots. (**C - E**) Enriched gene ontology terms of differentially expressed genes indicated on (A and B).

**Fig. S8**

**Fig. S8. Targeted degradation of AR regulates the expression of DNA replication genes in MFM-223 cells.** (**A**) Heat map of relative mRNA expression of *PCNA* and *ORC6* in MFM-223 cells treated with 100 nM HP518 or 1 μM HC-1119 versus vehicle for 24 and 48 h. n = 3. (**B**) Immunoblots of AR, PCNA, and ORC6 in MFM-223 cells treated with 100 nM HP518 or 1 μM HC-1119 versus vehicle for 24 and 48 h.

**Fig. S9**

**Fig. S9. The expression of DNA replication genes are independent on androgens.** (**A**) Relative mRNA expression of *PCNA* and *ORC6* in MDA-MB-453 cells cultured in medium supplemented with charcoal-stripped FBS (CSS) in the presence or absence of R1881 for 24 h and 48 h. n = 3. (**B**) Relative mRNA expression of *PCNA* and *ORC6* in MDA-MB-453 cells treated with the indicated treatments versus vehicle for 24 h and 48 h in the presence or absence of 100 nM R1881. n = 3.

**Fig. S10**

**Fig. S10. Motif analysis from AR CUT&Tag.** Top depleted motifs of chromatin sites with reduced accessibility in the HP518-treated cells relative to the vehicle-treated cells. n = 2.

**Fig. S11**

**Fig. S11. Transcription factors FoxA1 and GATA3 are required for the survival of AR-positive TNBC cells.** Proliferation analysis of MDA-MB-453 (A), MFM-223 (B), and SUM159PT (C) cells transduced with mock, control, *FoxA1*, or *GATA3* siRNAs. Data expressed as mean ± SD (n = 3).

**Fig. S12**

**Fig. S12. Targeted degradation of AR by HP518 impairs the interaction between AR and FoxA1/ GATA3.** Quantifications (Upper) and representative PLA images (Lower) of AR:FoxA1 (**A**), AR:GATA3 (**B**), and FoxA1:GATA3 (**C**) interactions in MDA-MB-453 cells treated with vehicle or 100 nM HP518 for 8 h. n = 3. PLA signals were quantified from at least 40 different images and showed as box and whisker plots display the median PLA signals per cell with whisker incorporating min-to-max of all data. Significance was analyzed using unpaired two-tail student’s *t* test (ns: not significant; ****P* < 0.001). Scale bars, 10 μm.

**Fig. S13**

**Fig. S13. DNA binding activities of recombinant AR DBD, FoxA1 FL, and GATA3 FL proteins.** (**A**) Sequences of the positive control DNA probes for AR DBD, FoxA1 FL, and GATA3 FL. (**B – D**) EMSA analysis of the binding between positive control DNA probes with AR DBD (B), FoxA1 FL (C), and GATA3 FL (D) proteins. The biotinylated DNA probes were synthesized harboring the classical binding motif and the unbiotinylated probes served as competitors. The binding was analyzed by immunoblotting with a biotin antibody.

**Fig. S14**

**Fig. S14. *In vitro* evaluation of BR5009 and LD1-2009-361825 models.** (**A**) Representative AR IHC images in the LD1-2009-361825 patient samples. Scale bar, 30 μm. (**B, C**) Combinatorial efficacy and synergy analysis of HP518 in the BR5009 (B) and LD1-2009-361825 (C) cells. HP518 was combined with either alpelisib or paclitaxel in BR5009 (left panel) and LD1-2009-361825 (right panel) cells for 7 days of treatment. The combination effects were evaluated using the Highest Single Agent (HSA) model in Combenefit. Synergy scores were calculated by comparing observed responses with the expected effects defined as the maximal response of either single agent and visualized as a synergy map projected onto the dose–response (D–R) surface. n = 3.

**Fig. S15**

**Fig. S15. Representative AR IHC images in the four AR-positive TNBC PDO samples.** Scale bar, 50 μm.

**Fig. S16**

**Fig. S16. HP518 impairs cell viability in the four AR-positive TNBC PDO models.** Dose-response curves of four AR-positive TNBC PDO models (0112201111, 0112301500, 0112302335, and 0112302288) treated with HP518, paclitaxel, or sacituzumab govitecan for 7 days. Data expressed as mean ± SD (n = 2).

**Table S1.**

Relative gene expression in MDA-MB-453 from RNA-Seq.

**Table S2.**

Pearson correlation coefficients between *AR* and DNA replication-related genes in AR-positive TNBC patients.

**Table S3.**

Gene effects (Chronos scores) of *PCNA*, *ORC6*, *FKBP5*, and *POLD3* among 1,100 cancer cell lines from the DepMap.

Tables S1 to S3 will be provided separately.
